# Immunoinformatics-driven multi-epitope vaccine design targeting PSMA, STEAP1, and B7H3 for prostate cancer

**DOI:** 10.3389/fmed.2026.1716345

**Published:** 2026-03-05

**Authors:** Stefanus Vicky Bernhard Elisa Runtunuwu, Trina Ekawati Tallei, Hyo Jeong Kim, Moon Nyeo Park, Ismail Celik, Burak Kirilmaz, Grace Lendawati Amelia Turalaki, Fatimawali Fatimawali, Lydia Estelina Naomi Tendean, Martha Marie Kaseke, Dionisius Rafael Makawaehe, Elne Vieke Rambi, Bonglee Kim

**Affiliations:** 1 Medical Education Study Program, Faculty of Medicine, Sam Ratulangi University, Manado, Indonesia; 2 Department of Biology, Faculty of Medicine, Sam Ratulangi University, Manado, Indonesia; 3 Department of Biology, Faculty of Mathematics and Natural Sciences, Sam Ratulangi University, Manado, Indonesia; 4 Department of Pathology, College of Korean Medicine, Kyung Hee University, Seoul, Republic of Korea; 5 Korean Medicine-Based Drug Repositioning Cancer Research Center, College of Korean Medicine, Kyung Hee University, Seoul, Republic of Korea; 6 Department of Pharmaceutical Chemistry, Faculty of Pharmacy, Erciyes University, Kayseri, Türkiye; 7 Pharmacy Study Program, Faculty of Mathematics and Natural Sciences, Sam Ratulangi University, Manado, Indonesia; 8 Department of Anatomy and Histology, Faculty of Medicine, Sam Ratulangi University, Manado, Indonesia; 9 Department of Medical Laboratory Technology, Politeknik Kesehatan Kemenkes Manado, Manado, Indonesia

**Keywords:** computational vaccine design, immunoinformatics, immunotherapy, multi-epitope, precision medicine, prostate cancer, tumor-associated antigens

## Abstract

**Introduction:**

Prostate cancer remains a major global health challenge, necessitating precision immunotherapeutic strategies tailored to tumor-associated antigens. This study aimed to design a multi-epitope peptide vaccine targeting prostate-specific membrane antigen (PSMA), six-transmembrane epithelial antigen of prostate 1 (STEAP1), and B7-H3, three biomarkers strongly associated with prostate cancer progression.

**Methods:**

A multi-layered immunoinformatics-driven approach was employed, integrating epitope prediction, antigenicity and immunogenicity assessment, allergenicity and toxicity screening, population coverage analysis, molecular docking, and molecular dynamics simulations. Selected epitopes were assembled into a vaccine construct using appropriate adjuvants and linkers to enhance immune activation and structural stability.

**Results:**

The designed vaccine construct demonstrated extensive global HLA allele coverage (97.51%), strong binding affinity to B-cell receptors, MHC molecules, and favorable structural stability during molecular dynamics simulations.

**Discussion:**

These findings suggest that the proposed multi-epitope vaccine represents a promising immunotherapeutic candidate for prostate cancer and warrants further experimental validation.

## Graphical Abstract

**Figure:**
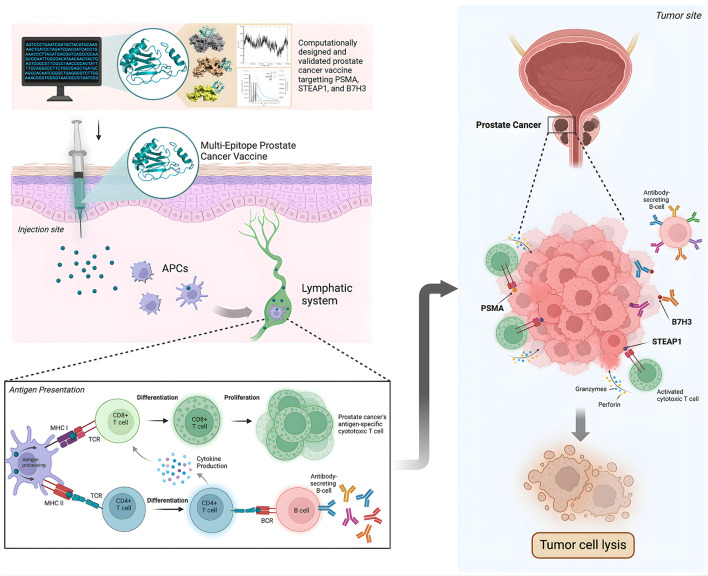
This study presents a computationally designed multi-epitope peptide vaccine targeting prostate cancer-associated antigens PSMA, STEAP1, and B7H3. The top-left section illustrates the bioinformatics pipeline, including vaccine construction, molecular docking, molecular dynamics, and immune simulation analyses, which guide vaccine design. Upon administration, the vaccine is processed by antigen-presenting cells (APCs) and transported through the lymphatic system, triggering an adaptive immune response. The differentiation and proliferation of CD8+ cytotoxic T cells and CD4+ helper T cells enhance tumor recognition, while B cells produce antigen-specific antibodies. Activated cytotoxic T cells release perforin and granzymes, leading to tumor cell lysis. This immunoinformatics-driven approach supports the development of targeted prostate cancer immunotherapy with potential clinical applications. Created using BioRender.com.

## 1 Introduction

Prostate cancer continues to be a significant global health challenge, impacting millions of men worldwide. It is the second most prevalent cancer among men and the fourth most commonly diagnosed malignancy overall, with nearly 1.4 million new cases and approximately 400,000 deaths reported in 2022 (1). One in eight men is estimated to be diagnosed with prostate cancer during their lifetime (2). Currently, treatment modalities such as prostatectomy, radiotherapy, and androgen deprivation therapy are broadly applied across the various stages of the disease (3). However, these therapies are associated with significant side effects, such as sexual dysfunction, fatigue, and increased risk of cardiovascular disease, which impact patients' quality of life (4). Moreover, in advanced stages, prostate cancer frequently progresses to castration-resistant prostate cancer (CRPC), a condition characterized by poor prognosis and limited treatment options (3, 5, 6). Consequently, there is an urgent need for novel therapeutic strategies that offer improved efficacy while minimizing adverse effects.

As one of the five main pillars of cancer therapy, immunotherapy harnesses the patient's immune system to recognize and eliminate tumor cells (7). Immunotherapeutic approaches include immune checkpoint inhibitors, immune system modulators, and cancer vaccines (7–9). Sipuleucel-T, an autologous cellular immunotherapy, has been approved by the Food and Drug Administration (FDA) for the treatment of advanced prostate cancer, demonstrating the potential of vaccine-based strategies (10, 11). Despite this success, current vaccine approaches face challenges due to the high heterogeneity of prostate cancer cells, which limits the ability of single-epitope vaccines to provide broad and durable immune responses (12–15).

Given the marked phenotypic and molecular heterogeneity of prostate cancer, precision medicine has emerged as a critical paradigm for developing tailored therapies. In this context, multi-epitope vaccines represent a promising precision immunotherapy strategy by simultaneously targeting multiple antigenic determinants. This approach enhances both humoral and cellular immune responses, reduces the risk of immune escape, and allows for optimization with adjuvants to improve immunogenicity and durability (16). Recent studies suggest that plant-derived bioactive compounds possess immunomodulatory properties and may serve as potential adjuvants in vaccine formulations. For instance, *Annona muricata* extracts have been shown to modulate key inflammatory cytokines and enhance tumor suppressor gene expression, indicating their potential role in cancer immunotherapy (17). As vaccine efficacy heavily depends on epitope selection, identifying highly immunogenic epitopes capable of eliciting robust B- and T-cell responses remains a critical priority (18). Combining precise epitope selection with optimized adjuvants could significantly enhance prostate cancer vaccine effectiveness.

Prostate-specific membrane antigen (PSMA), six-transmembrane epithelial antigen of the prostate 1 (STEAP1), and B7H3 (CD276, an immune checkpoint molecule of the B7 family that is overexpressed in prostate cancer and promotes immune evasion) were selected as vaccine targets based on their consistently high expression in primary and advanced prostate cancer and their strong potential as immunogenic antigens (19–23). Although other clinically relevant targets such as TROP2 and DLL3 are also implicated in prostate cancer biology (24), PSMA, STEAP1, and B7H3 were prioritized because they exhibit superior tumor specificity, possess well-characterized extracellular domains suitable for epitope design, and have substantial preclinical and translational evidence supporting their use in immunotherapy. Their complementary biological functions [PSMA in proliferation and neovascularization (25), STEAP1 in metabolic and intercellular signaling (26), and B7H3 in immune evasion (27)] provide a mechanistically diverse antigen set that strengthens a multi-antigen vaccine strategy aimed at reducing immune escape and addressing tumor heterogeneity. In contrast, other prostate cancer–associated targets, including TROP2, DLL3, and cancer–testis antigens such as SSX-2, were not prioritized due to limitations related to tumor specificity, heterogeneous or subtype-restricted expression patterns, and the absence of well-defined extracellular domains suitable for antibody-accessible epitope targeting (24).

Accordingly, the aim of this study was to employ an immunoinformatics-driven approach to design a peptide-based multi-epitope vaccine targeting PSMA, STEAP1, and B7-H3 as precision immunotherapy candidates for prostate cancer. To achieve this, a multi-layered computational pipeline was implemented that integrates antigen retrieval from curated databases, epitope prediction, and systematic screening to ensure rigorous and reproducible candidate selection. Linear B-cell epitopes were predicted using established sequence-based prediction algorithms, while cytotoxic and helper T-cell epitopes were identified using NetMHCpan and NetMHCIIpan based on both binding affinity and eluted-ligand models. Candidate epitopes were further evaluated for antigenicity, non-toxicity, non-allergenicity, and global population coverage, followed by structural modeling, molecular docking, and molecular dynamics simulations to assess construct stability and receptor compatibility. B-cell and T-cell epitopes were prioritized using distinct biological criteria, reflecting differences in antibody accessibility and endogenous antigen processing pathways.

## 2 Materials and methods

**Figure 1:**
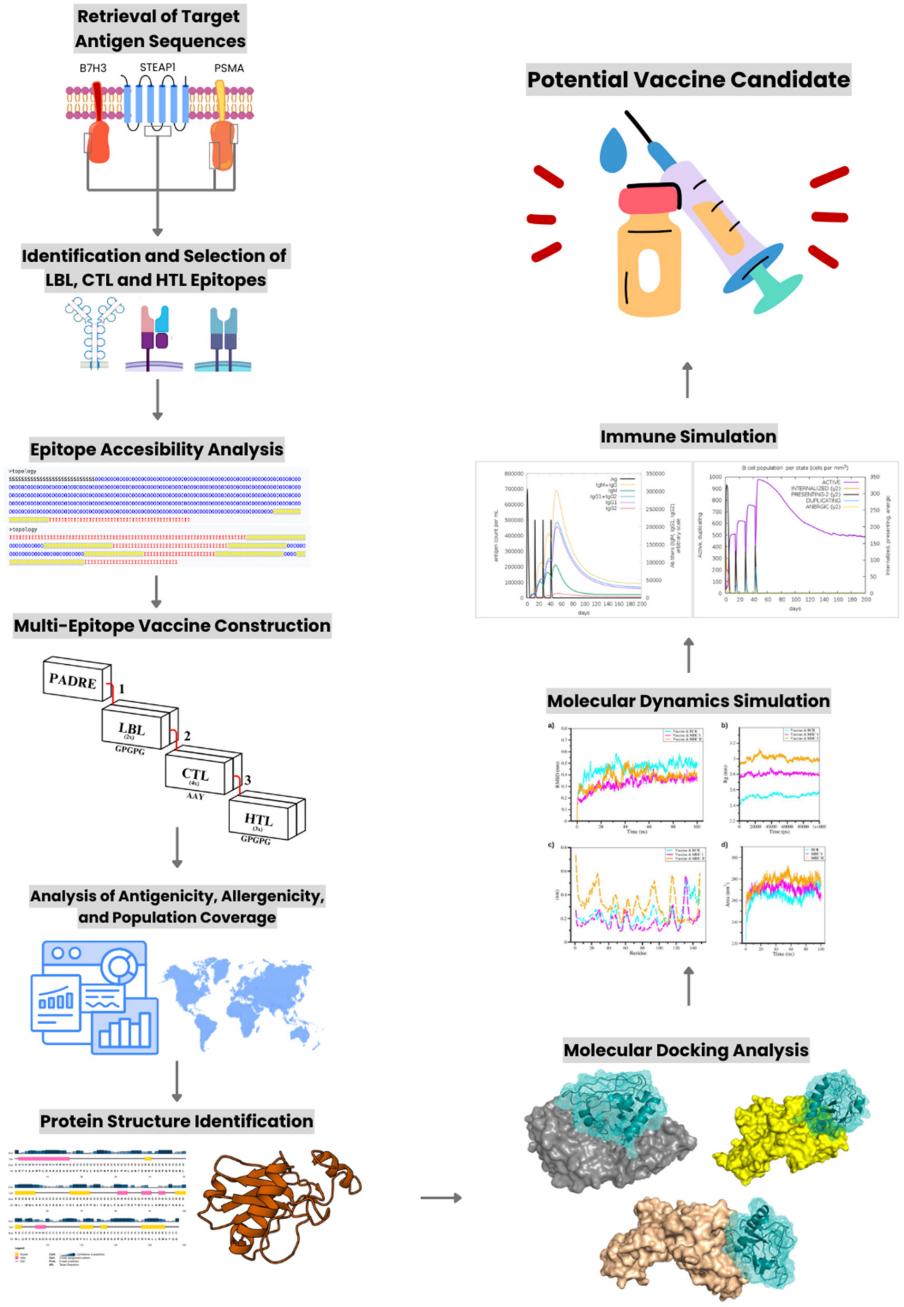
Overall workflow of the immunoinformatics pipeline used for multi-epitope vaccine construction. LBL, linear B-cell epitopes; CTL, cytotoxic T-lymphocyte epitopes (MHC class I); HTL, helper T-lymphocyte epitopes (MHC class II); PADRE, Pan-DR epitope adjuvant. The workflow includes antigen retrieval, epitope prediction, accessibility analysis, vaccine assembly, antigenicity/allergenicity/toxicity assessment, population coverage analysis, structural modeling, molecular docking, molecular dynamics simulation, and immune simulation leading to the identification of a potential vaccine candidate. Created with Canva.com.

The methods depicted in Figure 1 began with retrieving sequences of the target antigens, followed by identifying LBL, cytotoxic T-lymphocyte (CTL), and helper T-lymphocyte (HTL) epitopes. These epitopes were then assembled into a multi-epitope vaccine construct, whose immunogenic performance was predicted via *in silico* immune simulations. The construct was further assessed for antigenicity, allergenicity, and population coverage. Subsequently, molecular dynamics simulations (MDS) were employed to evaluate structural stability, and molecular docking analyses confirmed binding interactions. The final outcome was a refined vaccine candidate with strong potential for further development.

### 2.1 Retrieval of target antigen sequences

The full-length amino acid sequences of prostate cancer-associated proteins were retrieved from the NCBI Protein database (https://www.ncbi.nlm.nih.gov/) and UniProt (https://www.uniprot.org/) for subsequent epitope prediction and vaccine design (28, 29). Selection criteria included proteins that are overexpressed in prostate cancer cells and have established roles in tumor progression and immune evasion. Specifically, STEAP1 and PSMA sequences were acquired from NCBI using GenBank Accession IDs NP_036581.1 and AAA60209.1, respectively, while the B7H3 sequence was retrieved from UniProt (UniProt ID: Q5ZPR3).

### 2.2 Selection and analysis of linear B-cell epitopes

Linear B-cell epitopes were identified using the IEDB server (http://tools.iedb.org/bcell/) with the BepiPred-2.0 method, applying a default threshold of 0.5 to classify residues as potential epitopes. This method was selected due to its improved performance over previous sequence-based predictors, leveraging a machine-learning approach trained on epitope data derived from antigen-antibody complex structures (30). Based on B-cell receptor binding efficiency, the optimal peptide length range for effective immune recognition is 5–20 amino acids. Epitopes with scores above the threshold were selected for further evaluation.

Following epitope prediction, selected epitopes underwent antigenicity and toxicity screening to ensure suitability for vaccine formulation. Antigenicity was assessed using the VaxiJen 2.0 server, with a tumor antigen model threshold of 0.5 (31). Epitopes scoring above this threshold were classified as antigenic and retained for further analysis. Toxicity was evaluated using the ToxinPred server, employing an SVM-based classification model with default settings. Epitopes predicted as non-toxic were retained for further analysis. All analyses were performed using default parameters unless otherwise specified to ensure reproducibility.

### 2.3 Screening of T-cell epitopes

T-cell epitope identification was conducted using the IEDB Analysis Resource (http://tools.iedb.org/mhci/ and http://tools.iedb.org/mhcii/), utilizing both binding affinity (BA) and eluted ligand (EL) models for MHC class I and MHC class II predictions, ensuring the identification of epitopes that not only exhibit strong predicted binding affinity to MHC molecules but also have experimental support from eluted ligand datasets. Specifically, NetMHCIpan 4.1 BA and NetMHCIpan 4.1 EL were used for MHC class I epitope predictions, while NetMHCIIpan 4.1 BA and NetMHCIIpan 4.1 EL were used for MHC class II predictions. These tools leverage artificial neural networks trained on both binding affinity measurements and mass spectrometry-eluted ligand data, providing improved predictive accuracy compared to conventional sequence-based models (32, 33).

For MHC class I epitope prediction, peptides were evaluated based on their predicted binding affinity to MHC class I molecules. Selection criteria included an IC_50_ value of < 50 nM and a percentile rank ≤ 0.06, ensuring the identification of high-affinity binders (32–34). These stringent thresholds were chosen because MHC class I molecules present peptides derived from intracellular proteins to CD8^+^ T cells, where only the highest-affinity peptides are likely to trigger a strong immune response (35). The screening was performed using a diverse set of human HLA class I alleles, as proposed by Weiskopf et al. (36), to ensure broad population coverage (Table 1). Following this, the selected epitopes underwent further antigenicity assessment using the VaxiJen v2.0 server (tumor model threshold: 0.5) and toxicity evaluation using the ToxinPred server. Only epitopes predicted to be highly antigenic and non-toxic were retained. To optimize the vaccine construct, redundant epitopes were filtered out, and the final selection was made based on strong antigenicity scores and broad HLA binding potential.

**Table 1:** Alleles used for T-cell epitope identification.

**Epitopes**	**HLA allele**
HLA class I	A^*^01:01, A^*^02:01, A^*^02:03, A^*^02:06, A^*^03:01, A^*^11:01, A^*^23:01, A^*^24:02, A^*^26:01A^*^30:01, A^*^30:02, A^*^31:01, A^*^32:01, A^*^33:01, A^*^68:01, A^*^68:02, B^*^07:02, B^*^08:01, B^*^15:01, B^*^35:01, B^*^40:01, B^*^44:02, B^*^44:03, B^*^51:01, B^*^53:01, B^*^57:01, B^*^58:01
HLA class II	DRB1^*^01:01, DRB1^*^03:01, DRB1^*^04:01, DRB1^*^04:05, DRB1^*^07:01, DRB1^*^08:02, DRB1^*^09:01, DRB1^*^11:01, DRB1^*^12:01, DRB1^*^13:02, DRB1^*^15:01, DRB3^*^01:01, DRB3^*^02:02, DRB4^*^01:01, DRB5^*^01:01, DQA1^*^05:01/DQB1^*^02:01, DQA1^*^05:01/DQB1^*^03:01, DQA1^*^03:01/DQB1^*^03:02, DQA1^*^04:01/DQB1^*^04:02, DQA1^*^01:01/DQB1^*^05:01, DQA1^*^01:02/DQB1^*^06:02, DPA1^*^02:01/DPB1^*^01:01, DPA1^*^01:03/DPB1^*^02:01, DPA1^*^01:03/DPB1^*^04:01, DPA1^*^03:01/DPB1^*^04:02, DPA1^*^02:01/DPB1^*^05:01, DPA1^*^02:01/DPB1^*^14:01

For MHC class II epitope prediction, NetMHCIIpan 4.1 was used to identify peptides with strong binding potential to MHC class II molecules. Epitopes were selected based on an IC_50_ value of < 500 nM and a percentile rank ≤ 0.1, which are slightly less stringent than MHC class I criteria (33, 34). This is because MHC class II molecules present peptides derived from extracellular or endocytosed proteins to CD4^+^ T cells, which typically accommodate a more diverse range of peptide affinities compared to MHC class I (35). To ensure broad population coverage, the screening was performed using a set of 26 globally common HLA class II alleles, selected based on the HLA reference set proposed by Greenbaum et al. (37) (Table 1). Those meeting these criteria were further assessed for antigenicity and toxicity, following the same approach as MHC class I epitopes. To avoid excessive vaccine sequence length, overlapping core peptides were excluded. Additionally, epitopes restricted to a single HLA allele with a percentile rank of 0.21 were also excluded from further analysis. The integration of both binding affinity and eluted ligand models ensured the selection of high-affinity, antigenic, and experimentally relevant T-cell epitopes, providing a robust foundation for the subsequent vaccine formulation process.

### 2.4 Extracellular domain analysis and epitope accessibility

B7H3, PSMA, and STEAP1 are transmembrane proteins with distinct functions. B7H3 and PSMA each have a single transmembrane domain, whereas STEAP1 is an ion transporter protein with six transmembrane domains. To determine the extracellular regions of these plasma membrane proteins, CCTOP (https://cctop.ttk.hu/) was used for topology prediction. CCTOP was selected due to its superior accuracy compared to single-method predictors, such as TMHMM and Phobius. It integrates ten different topology prediction algorithms with a Hidden Markov Model-based consensus approach, improving transmembrane region delineation and reducing false positives (38).

CCTOP predictions were generated using default parameters. Extracellular domains were defined as surface-exposed regions highlighted in blue in the consensus topology output, in contrast to transmembrane segments shown in red and cytoplasmic regions shown in yellow. All predicted extracellular loops were considered for each protein, including N- or C-terminal domains when classified as extracellular. These regions served as the basis for selecting epitopes accessible to immune recognition (39).

Following topology annotation, epitope accessibility was evaluated by mapping previously identified T-cell and B-cell epitopes onto the predicted extracellular regions. Epitopes were aligned to their corresponding protein sequences, and their positions were compared with the CCTOP-predicted topology. Only epitopes located entirely within extracellular loops were classified as accessible. Epitopes overlapping with transmembrane or cytoplasmic segments, even partially, were excluded to ensure that only surface-exposed antigenic sites were prioritized. This filtering step was essential to identify epitopes that can be recognized by components of the immune system and are suitable for antibody-mediated targeting or vaccine development (40, 41).

### 2.5 Finalization of epitope candidates and population coverage analysis

Potential epitopes that met the initial screening criteria and were predicted to be surface-exposed based on prior analysis were further refined based on their highest immunogenicity scores and broadest population coverage. Only the peptide with the highest immunogenicity score from each target antigen (PSMA, STEAP1, and B7H3) was chosen for each receptor type (BCR, MHC I, and MHC II). This selection process ensured an optimized yet concise multi-epitope vaccine construct.

To further assess the potential effectiveness of the selected epitopes in a real-world setting, population coverage analysis was conducted. Since HLA allele frequencies vary significantly across ethnic and regional populations (42), this analysis was crucial to ensure that the vaccine would provide immune coverage for a broad range of individuals worldwide. Population coverage analysis was performed using the IEDB Population Coverage Analysis tool (http://tools.iedb.org/population/) (43). The analysis was conducted simultaneously for both MHC class I and class II epitopes. Sixteen geographical regions were selected for evaluation with default parameters.

### 2.6 Multi-epitope vaccine construction and analysis of antigenicity, allergenicity, and secondary structure of the vaccine candidate

The final epitopes were assembled together with an adjuvant and linkers into a single multi-epitope vaccine sequence. PADRE was selected to provide broad CD4^+^ T-helper stimulation across diverse HLA class II alleles with minimal sequence variability (44); although protein-based adjuvants such as the C-terminal region of Mycobacterium tuberculosis HSP70 are commonly used in vaccine design, they were not incorporated here to avoid increased construct complexity and potential off-target innate immune activation beyond the scope of this epitope-centric strategy. To ensure proper separation between epitopes and optimize antigen presentation, specific linkers were used. EAAAK was used to connect the adjuvant to B-cell epitopes, ensuring structural stability and reducing unfavorable interactions between adjacent domains (45). GPGPG was used to connect B-cell epitopes, to link MHC class II epitopes, and to connect B-cell epitopes with MHC class I epitopes, improving antigen processing efficiency and enhancing immune recognition (46). AAY was used to connect MHC class I epitopes and to link MHC class I epitopes with MHC class II epitopes, optimizing proteasomal cleavage and enhancing epitope presentation (47, 48). The final vaccine construct was manually assembled by linking the selected adjuvant, epitopes, and linkers in a sequential order.

The antigenicity of the newly constructed vaccine was re-evaluated with standard parameters using the VaxiJen v2.0 server under the tumor model (31), which is optimized for identifying cancer-associated antigens. The allergenicity of the multi-epitope vaccine was assessed using the AllerTop v2.1 server (https://www.ddg-pharmfac.net/allertop_test/) and the AlgPred server (http://crdd.osdd.net/raghava/algpred/), which integrate various approaches to predict the allergenic potential of proteins based on amino acid composition and Immunoglobulin E (IgE) epitope prediction (49, 50).

The secondary structure of the newly formulated vaccine sequence was predicted using two servers, SOPMA (https://npsa.lyon.inserm.fr/) and PSIPRED (http://bioinf.cs.ucl.ac.uk/psipred). SOPMA was selected for secondary structure prediction due to its statistically optimized algorithm, which achieves 69%−72% accuracy in classifying secondary structure elements (51). PSIPRED was additionally used as it incorporates evolutionary position-specific scoring matrices (PSSM) and a two-layer neural network, achieving >76% accuracy in predicting alpha-helices, beta-sheets, and coil structures (52–55).

### 2.7 Modeling, 3D visualization, and validation of the vaccine structure

The I-TASSER server (Iterative Threading Assembly Refinement; https://zhanggroup.org/I-TASSER/) was used to predict the three-dimensional structure of the vaccine. I-TASSER was selected due to its ability to generate accurate full-length protein models by integrating template-based modeling with ab initio structure prediction (56).

The models generated by I-TASSER were ranked based on their C-score, a confidence score that predicts the quality of the modeled structure based on the significance of threading alignments and the convergence of structural assembly simulations (57). The model with the highest C-score, indicating the most reliable prediction, was selected for further refinement. The selected model was further refined using the GalaxyRefine2 server (https://galaxy.seoklab.org/cgi-bin/submit.cgi?type=REFINE2), which improves structural accuracy by optimizing atomic-level interactions and reducing steric clashes to enhance the overall model reliability (58).

Validation of the refined model was performed using the PROCHECK server (https://www.ebi.ac.uk/thornton-srv/software/PROCHECK/) by analyzing the Ramachandran plot, which assesses backbone torsion angles (phi, ϕ and psi, ψ) to determine structural quality (59). The percentage of residues in favored, allowed, and disallowed regions was evaluated to confirm the model's reliability.

A comparison between the initial model generated by I-TASSER and the refined model from GalaxyRefine2 was visualized using the MolStar viewer (https://molstar.org/viewer/). The final vaccine model, including epitopes, adjuvants, and linkers, was further visualized using PyMol to examine secondary and tertiary structures, epitope positioning, and overall structural features.

### 2.8 Molecular docking and protein interaction analysis

Receptor preparation was performed using PyMol to remove non-essential molecules, such as water, glycerol, and native ligands. These molecules were eliminated to prevent interference with docking simulations, ensuring accurate binding pocket conformation and molecular interaction calculations. Receptor structures were retrieved from the Protein Data Bank (PDB) with the following PDB IDs: 5IFH for B-cell receptor (BCR), 3BO8 for MHC class I, and 4I5B for MHC class II.

The modeled 3D structure of the vaccine protein was docked with B-cell, MHC I, and MHC II receptors using the ClusPro 2.0 server (https://cluspro.bu.edu). ClusPro performed docking by generating multiple orientations of the vaccine-receptor complex, which were then scored based on electrostatic interactions, Van der Waals forces, and solvation energy. Docking was conducted using ClusPro's Balanced mode, which optimally weighs electrostatics and Van der Waals forces to achieve the most physiologically relevant binding conformation. The docking poses were ranked, and the final model was selected based on the lowest binding energy score and the biological relevance of the binding interface. ClusPro was selected due to its high reliability for protein-protein docking, its ability to use Fast Fourier Transform (FFT) for rapid surface analysis, and its experimentally validated docking accuracy (60–64).

Binding affinity and dissociation constant (*K*_d_) analysis were performed using the PRODIGY server (https://wenmr.science.uu.nl/prodigy/), which predicts the stability of protein-protein interactions by calculating Gibbs free energy (Δ*G*) and estimating binding affinity based on atomic contact information. For this analysis, the temperature was set to 37 °C (310 K) to reflect human physiological conditions (65). Protein interaction interfaces resulting from docking were analyzed using PDBsum (https://www.ebi.ac.uk/thornton-srv/databases/pdbsum/). The vaccine-receptor complexes were visualized using PyMol (66). Allele-specific epitope–HLA was not performed in this study. Instead, global HLA representation of the selected epitopes was evaluated using population coverage analysis, while molecular docking focused on assessing the structural compatibility and stability of the final vaccine construct.

### 2.9 Molecular dynamics simulation

The system was prepared using the CHARMM-GUI online server due to its compatibility with efficient protein-water simulations and standard force fields, and simulations were performed using the AMBER ff14SB force fields in GROMACS version 2023 (GROMACS Development Team, Stockholm, Sweden) (67). The AMBER ff14SB force field was used due to its proven reliability in protein modeling (68), while the TIP3P water model was selected for its advanced accuracy in reproducing hydration properties (69). The system was neutralized with 0.15 M KCl to maintain ionic equilibrium, and the temperature and pressure were set to 310 K and 1 bar, respectively, to reflect standard human body conditions.

Although previous vaccine modeling studies have shown that even a 50-ns timeframe is sufficient to observe the early-stage conformational dynamics and stability of peptide-receptor interactions, a 100-ns simulation was conducted for a clearer and more comprehensive analysis (70–72). To evaluate stability, structural fluctuations and binding interactions were analyzed using root mean square deviation (RMSD), radius of gyration (Rg), solvent accessible surface area (SASA), and root mean square fluctuation (RMSF). These metrics provided information about the overall structural integrity, compactness, solvent exposure, and molecular interactions of the vaccine structure during the simulation (73–76).

### 2.10 Immune simulation

Following structural validation, immune response prediction was performed using C-ImmSim (https://kraken.iac.rm.cnr.it/C-IMMSIM/), an agent-based modeling tool designed to simulate antigen interactions with immune cells, epitope binding to MHC molecules, and immune memory formation (77). To simulate a clinically relevant vaccination strategy, four vaccine doses were administered at 2-week intervals (78), corresponding to time steps 1, 43, 85, and 127, where each time step represents 8 h (79). This schedule was designed to ensure consistent antigen exposure and facilitate the progressive activation and maturation of immune responses, enabling the observation of both short-term adaptive immunity and long-term immune memory formation (80). All other simulation parameters in C-ImmSim were used at default settings unless otherwise specified to ensure consistency and reproducibility of results. The simulation was conducted under the assumption of an ideal human immune system, optimal epitope exposure, and simplified biological conditions. This approach provides an initial evaluation of vaccine immunogenicity (81).

## 3 Results

### 3.1 Characterization of prostate cancer-associated antigens

The amino acid sequences of the three selected prostate cancer-associated antigens, B7H3, STEAP1, and PSMA, were analyzed. The respective protein lengths are 533, 339, and 750 amino acids. These antigens were chosen based on their high expression in prostate cancer cells, extracellular accessibility, and immunogenic potential. A multi-epitope approach utilizing these three antigens was evaluated, considering its potential to enhance immune recognition compared to targeting a single antigen. This strategy was designed to improve immune responses while reducing the risk of tumor immune escape.

### 3.2 Selected linear B-cell epitopes

A total of 54 linear B-cell epitopes were identified, comprising 23 from B7H3, eight from STEAP1, and 23 from PSMA. Based on B-cell receptor binding efficiency, 32 epitopes met the optimal length range of 5–20 amino acids for effective immune recognition. Further evaluation of antigenicity and toxicity led to the elimination of weakly antigenic or potentially harmful sequences. This refinement resulted in the selection of 14 highly antigenic and non-toxic B-cell epitopes, which were incorporated into the multi-epitope vaccine construct (Table 2).

**Table 2:** Final set of 14 highly antigenic and non-toxic linear B-cell epitopes derived from B7H3, STEAP1, and PSMA that were selected for incorporation into the multi-epitope vaccine construct.

**Prostate cancer antigen**	**Length (AA)**	**Peptide**	**Antigenicity**	**Toxicity**
B7H3	6	FPDLLA	1.7939	Non-toxic
		21	KQLVHSFTEGRDQGSAYANRT	0.8387	Non-toxic
		15	FAEGQDQGSAYANRT	0.7972	Non-toxic
		8	PVLQQDAH	0.7816	Non-toxic
		12	QPMTFPPEALWV	0.6147	Non-toxic
PSMA	13	QIPHLAGTEQNFQ	1.2403	Non-toxic
		17	IKSSNEATNITPKHNMK	1.0464	Non-toxic
		15	RTEDFFKLERDMKIN	0.9785	Non-toxic
		10	RLQDFDKSNP	0.9147	Non-toxic
		12	LHETDSAVATAR	0.8901	Non-toxic
		10	ANSIVLPFDC	0.8515	Non-toxic
STEAP1	15	IPSVSDSLTWREFHY	0.8094	Non-toxic
		16	GTKYKKFPHWLDKWML	0.7816	Non-toxic
		19	ILKIRHGWEDVTKINKTEI	0.6345	Non-toxic

### 3.3 Selected T-cell epitopes

T-cell epitopes were screened to identify peptide fragments capable of inducing immune responses through MHC class I and class II presentation. A total of 690 MHC class I epitopes were identified based on IC_50_ binding affinity and percentile rank criteria, using a diverse set of human HLA class I alleles to ensure broad genetic coverage. After refinement, 14 epitopes were retained, consisting of two from B7H3, seven from PSMA, and five from STEAP1. Among these, FPGIYDALF (PSMA) and RSYRYKLLNW (STEAP1) were included despite being restricted to a single HLA allele, as they showed the highest immunogenicity scores. The 14 selected MHC class I epitopes are summarized in Table 3.

**Table 3:** Final set of 14 high-affinity and immunogenic MHC class I T-cell epitopes derived from B7H3, STEAP1, and PSMA selected for inclusion in the multi-epitope vaccine construct.

**Prostate cancer antigen**	**Epitopes**	**Allele hit**	**IC_50_ (nM)**	**Antigenicity**	**Toxicity**
B7H3	MTFPPEALW	HLA-A^*^32:01	33.28	0.6275	Non-toxic
		HLA-B^*^57:01	4.85		Non-toxic
		HLA–B^*^58:01	2.37		Non-toxic
		HLA–B^*^53:01	27.54		Non-toxic
	AQLNLIWQL	HLA-A^*^02:06	3.26	0.7006	Non-toxic
		HLA-A^*^02:01	8.61		Non-toxic
PSMA	EIASKFSER	HLA-A^*^33:01	18.58	0.7496	Non-toxic
		HLA-A^*^68:01	7.76		Non-toxic
	ALFDIESKV	HLA-A^*^02:01	11.56	0.8125	Non-toxic
		HLA-A^*^02:03	6.0		Non-toxic
		HLA-A^*^02:06	31.2		Non-toxic
	AENIKKFLY	HLA-B^*^44:02	27.96	0.8552	Non-toxic
		HLA-B^*^44:03	34.19		Non-toxic
	SVYETYELV	HLA-A^*^68:02	17.93	0.8802	Non-toxic
		HLA-A^*^02:06	17.24		Non-toxic
	KVFRGNKVK	HLA-A^*^30:01	12.86	1.0112	Non-toxic
		HLA-A^*^03:01	39.72		Non-toxic
	KYADKIYSI	HLA-A^*^23:01	12.6	1.3426	Non-toxic
		HLA-A^*^24:02	9.97		Non-toxic
	FPGIYDALF	HLA-B^*^53:01	9.45	1.5015	Non-toxic
STEAP1	FLYTLLREV	HLA-A^*^02:03	2.18	0.5316	Non-toxic
		HLA-A^*^02:01	3.81		Non-toxic
		HLA-A^*^02:06	5.31		Non-toxic
	SELQHTQELF	HLA-B^*^44:02	26.01	0.8385	Non-toxic
		HLA-B^*^44:03	30.45		Non-toxic
	ATSHQQYFY	HLA-A^*^01:01	30.25	0.8464	Non-toxic
		HLA-A^*^30:02	48.08		Non-toxic
	IPSVSDSLTW	HLA-B^*^53:01	9.74	1.0273	Non-toxic
		HLA-B^*^58:01	4.41		Non-toxic
		HLA-B^*^57:01	22.94		Non-toxic
	RSYRYKLLNW	HLA-B^*^57:01	7.96	1.6931	Non-toxic

For MHC class II epitopes, 731 candidates were identified across 26 globally common HLA class II alleles to ensure broad population coverage. Based on IC_50_ binding affinity and percentile rank criteria, 21 epitopes were retained following antigenicity and toxicity screening. The top-scoring epitopes included NASLRLQRVRVADEGS, DRYVILGGHRDS, and LIFAWNKWIDIK, with antigenicity scores of 1.2529, 1.7466, and 1.0460, respectively. To maximize coverage while maintaining the vaccine construct within 200 amino acids, 11 epitopes recognized by multiple HLA alleles were selected. The final set of MHC class II epitopes is presented in Table 4.

**Table 4:** Final set of 11 high-affinity, antigenic, and non-toxic MHC class II epitopes derived from B7H3, STEAP1, and PSMA selected for incorporation into the multi-epitope vaccine construct.

**Prostate cancer antigen**	**Epitopes**	**IC_50_ (nM)**	**Antigenicity**	**Toxicity**
B7H3	ALWFCLTGALEVQ	51.26	0.6660	Non-toxic
		NASLRLQRVRVADEGS	32.10	1.2529	Non-toxic
		GNASLRLQRVRVA	179.46	0.5191	Non-toxic
PSMA	NYTLRVDCTPLMY	30.55	0.9264	Non-toxic
		RIYNVIGTLRGA	195.17	0.9996	Non-toxic
		KVFRGNKVKNAQ	358.41	1.1254	Non-toxic
		HPNYISIINEDGNE	189.62	1.3515	Non-toxic
		DRYVILGGHRDS	309.98	1.7466	Non-toxic
STEAP1	LHAIYSLSYPMRRSYR	11.70	0.6878	Non-toxic
		LIFAWNKWIDIK	56.30	1.0460	Non-toxic
		RSYRYKLLNWAYQQ	149.59	1.0531	Non-toxic

### 3.4 Extracellular domain analysis and epitope accessibility

After screening T-cell epitopes for high immunogenic potential, extracellular domain analysis was performed to determine the surface accessibility of B-cell epitopes, as antibody-mediated recognition requires exposed regions on the tumor cell surface. Topology assessment confirmed that B7H3 and PSMA each contain a single transmembrane domain, with most of their structures localized extracellularly. In contrast, STEAP1 was identified as a multipass transmembrane protein containing six transmembrane domains; therefore, only its three extracellular loops were evaluated as candidates for B-cell epitope selection due to their surface accessibility. Intracellular or transmembrane regions of all three antigens may still be processed and presented via MHC class I or II and therefore remain valid sources of T-cell epitopes.

Among the screened epitopes for B7H3 and PSMA, most of the B-cell epitope candidates were found to be extracellularly accessible for potential antibody recognition. However, three B-cell candidates (QPMTFPPEALWV from B7H3, LHETDSAVATAR from PSMA, and MTFPPEALW from B7H3) were located within transmembrane or cytoplasmic regions and were therefore excluded from B-cell consideration (Table 5). For STEAP1, topology analysis indicated that most candidate B-cell epitopes were not exposed on the cell surface, resulting in no STEAP1-derived B-cell epitopes being retained.

**Table 5:** Topology prediction of B7H3 and PSMA proteins.

**Protein segment**	**Prostate cancer antigen**
MLRRRGSPGMGVHVGAALGALWFCLTGALEVQVPEDPVVALVGTDATLCCSFSPEPGFSLAQLNLIWQLTDTKQLVHSFAEGQDQ GSAYANRTALFPDLLAQGNASLRLQRVRVADEGSFTCFVSIRDFGSAAVSLQVAAPYSKPSMTLEPNKDLRPGDTVTITCSSYQ GYPEAEVFWQDGQGVPLTGNVTTSQMANEQGLFDVHSILRVVLGANGTYSCLVRNPVLQQDAHSSVTITPQRSPTGAVEVQVPE DPVVALVGTDATLRCSFSPEPGFSLAQLNLIWQLTDTKQLVHSFTEGRDQGSAYANRTALFPDLLAQGNASLRLQRVRVADEGS FTCFVSIRDFGSAAVSLQVAAPYSKPSMTLEPNKDLRPGDTVTITCSSYRGYPEAEVFWQDGQGVPLTGNVTTSQMANEQGLFD VHSVLRVVLGANGTYSCLVRNPVLQQDAHGSVTITGQPMTFPPEALWVTVGLSVCLIALLVALAFVC WRKIKQSCEEENAGAEDQ DGEGEGSKTALQPLKHSDSKEDDGQEIA	B7H3
MWNLLHETDSAVATARRPRW LCAGALVLAGGFFLLGFLFGWFI KSSNEATNITPKHNMKAFLDELKAENIKKFLYNFTQIPHLAGT EQNFQLAKQIQSQWKEFGLDSVELAHYDVLLSYPNKTHPNYISIINEDGNEIFNTSLFEPPPPGYENVSDIVPPFSAFSPQGMPE GDLVYVNYARTEDFFKLERDMKINCSGKIVIARYGKVFRGNKVKNAQLAGAKGVILYSDPADYFAPGVKSYPDGWNLPGGGVQ RGNILNLNGAGDPLTPGYPANEYAYRRGIAEAVGLPSIPVHPIGYYDAQKLLEKMGGSAPPDSSWRGSLKVPYNVGPGFTGN FSTQKVKMHIHSTNEVTRIYNVIGTLRGAVEPDRYVILGGHRDSWVFGGIDPQSGAAVVHEIVRSFGTLKKEGWRPRRTILFA SWDAEEFGLLGSTEWAEENSRLLQERGVAYINADSSIEGNYTLRVDCTPLMYSLVHNLTKELKSPDEGFEGKSLYESWTKKS PSPEFSGMPRISKLGSGNDFEVFFQRLGIASGRARYTKNWETNKFSGYPLYHSVYETYELVEKFYDPMFKYHLTVAQVRGGMVF ELANSIVLPFDCRDYAVVLRKYADKIYSISMKHPQEMKTYSVSFDSLFSAVKNFTEIASKFSERLQDFDKSNPIVLRMMNDQL MFLERAFIDPLGLPDRPFYRHVIYAPSSHNKYAGESFPGIYDALFDIESKVDPSKAWGEVKRQIYVAAFTVQAAAETLSEVA	PSMA

Yellow denotes transmembrane regions and red denotes cytoplasmic domains. Extracellular segments were used for B-cell epitope selection, whereas intracellular and transmembrane regions were still eligible for MHC class I and II epitopes due to endogenous antigen processing.

Two STEAP1-derived T-cell epitopes, RSYRYKLLNW (MHC class I) and RSYRYKLLNWAYQQ (MHC class II), were located within extracellular loops and retained for further analysis (Table 6). Their selection was based on T-cell epitope criteria rather than membrane accessibility, as T-cell epitopes do not require extracellular localization. Extracellular positioning was noted only to describe their topological context, and epitopes deemed inaccessible were excluded solely from B-cell epitope selection.

**Table 6:** Topology distribution of the multi-pass transmembrane protein STEAP1.

**Protein segment**	**Domain type**
HPLATSHQQYF	Extracellular
RRSYRYKLLNWAYQQVQQNKEDAWIEHDVW	
DIKQ	
IAAIIASLTFLYTLLREVI	Transmembrane
YKIPILVINKVLPMVSITLLAL	
LLSFFFAVLHAIYSLSYPM	
RMEIYVSLGIVGLAILALL	
LGIVSLLLGTIHALIFAWNKWI	
FVWYTPPTFMIAVFLPIVVLIFKSILF	
MESRKDITNQEELWKMKPRRNLEEDDYLHKD TGETSMLKRPVLLHLHQTAHADEFDCPSELQ HTQELFPQWHLPIK	Cytoplasmic
VYLPGVIAAIVQLHNGTKYKKFPHWLDKW MLTRKQFG	
AVTSIPSVSDSLTWREFHYIQSK	
LPCLRKKILKIRHGWEDVTKINKTEICSQL	

### 3.5 Finalization of epitope candidates and population coverage analysis

To ensure vaccine efficiency, only the most immunogenic epitopes with the broadest population coverage were selected from each antigen for further processing (Table 7). For B-cell epitopes, only extracellularly accessible regions were retained, while T-cell epitopes were selected from both extracellular and intracellular regions, as intracellular fragments may be processed and presented by MHC class I and II. Although peptide vaccines do not have strict length limitations, selecting shorter epitopes improves manufacturability, cost-effectiveness, and biological processing efficiency, as shorter peptides are easier to synthesize, purify, and produce at scale.

**Table 7:** Final epitopes selected for multi-epitope vaccine construction.

**Immune receptor**	**Epitope**	**Prostate cancer antigen**
B-cell lymphocyte	FPDLLA	B7H3
		QIPHLAGTEQNFQ	PSMA
MHC class I	AQLNLIWQL	B7H3
		KYADKIYSI	PSMA
		FPGIYDALF	PSMA
		RSYRYKLLNW	STEAP1
MHC class II	NASLRLQRVRVADEGS	B7H3
		DRYVILGGHRDS	PSMA
		RSYRYKLLNWAYQQ	STEAP1

**Figure 2:**
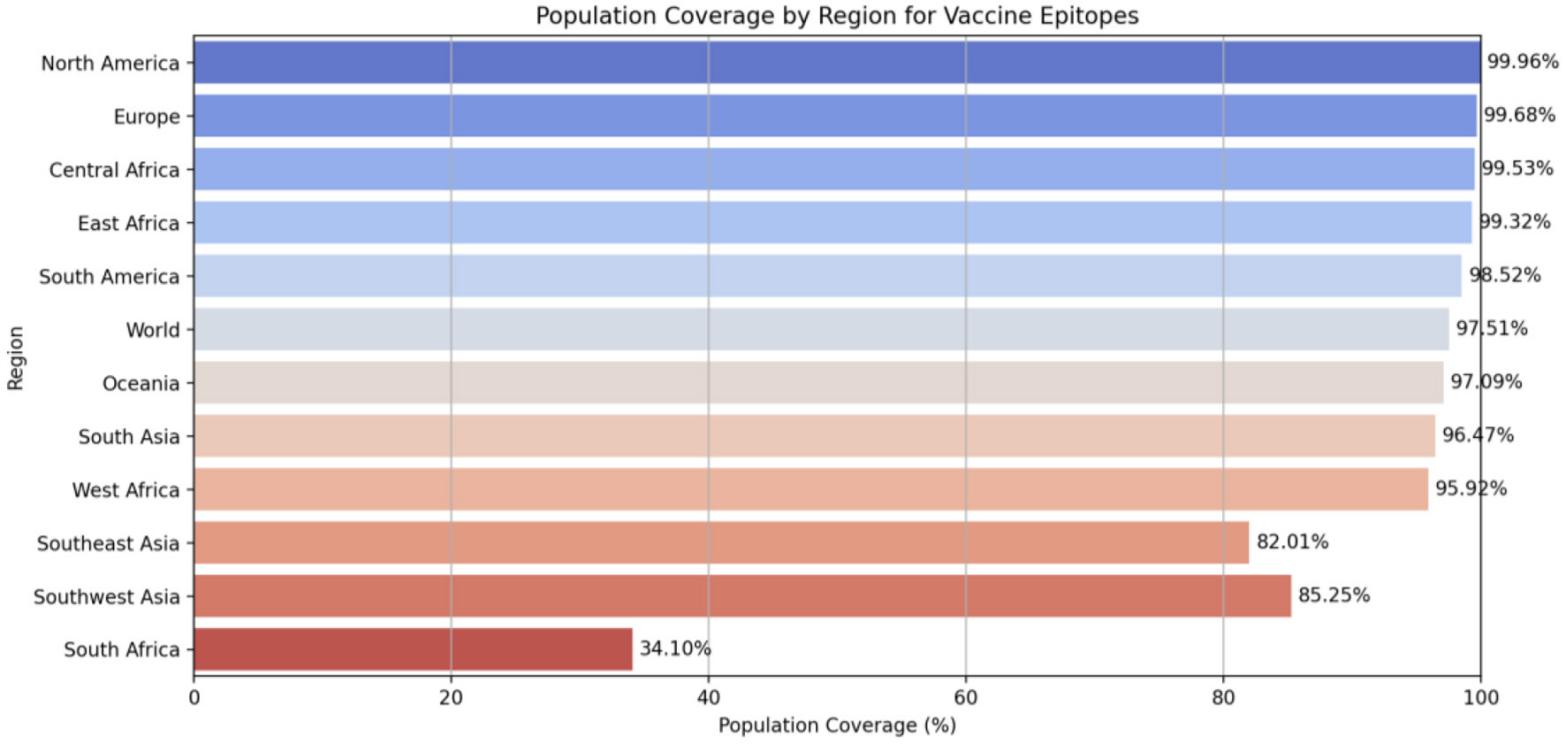
Population coverage analysis of combined MHC I and II epitopes.

The population coverage analysis (Figure 2) shows that the selected MHC class I and II epitopes achieved a global average coverage of 97.51%, indicating high inclusivity across diverse HLA alleles. North America, Europe, and Central Africa demonstrated near-complete coverage, with percentages exceeding 99%. In contrast, Southeast Asia, Southwest Asia, and South Africa exhibited lower coverage, at 82.01, 85.25, and 34.1%, respectively. These differences are likely due to regional variations in HLA allele distribution. Epitopes with limited population coverage were excluded to maximize global applicability. The final selection balances high immunogenicity with broad HLA allele representation, ensuring an optimal immune response across diverse populations.

### 3.6 Multi-epitope vaccine construction and analysis of antigenicity, allergenicity, and secondary structure of the vaccine candidate

**Figure 3:**
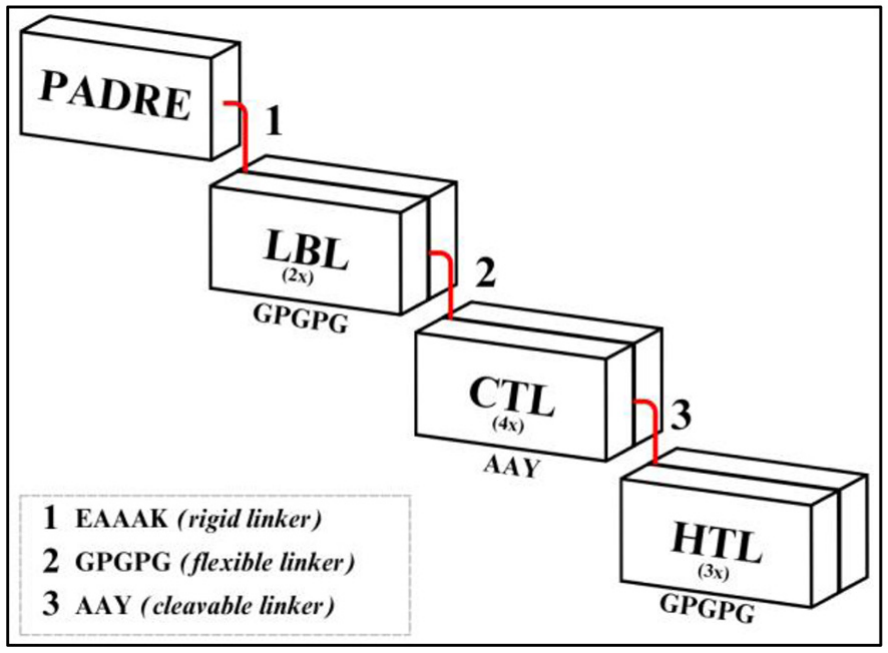
Schematic representation of the multi-epitope vaccine construct, highlighting Adjuvant, Linear B-Lymphocyte (LBL) epitopes, Cytotoxic T-Lymphocyte (CTL) epitopes, Helper T-Lymphocyte (HTL) epitopes and its linkers.

After finalizing the epitope selection based on immunogenicity and population coverage, the multi-epitope vaccine construct was designed. This involved assembling the selected epitopes with linkers and an adjuvant, followed by computational analyses to assess antigenicity, allergenicity, and structural stability. To enhance vaccine immunogenicity, a CD4^+^ T-helper epitope was added at the N-terminus to improve antigen presentation across multiple HLA-DR alleles. Linker sequences were incorporated to ensure structural integrity and efficient antigen processing. AAY linkers were used to connect MHC class I epitopes and bridge MHC class I with class II epitopes. GPGPG linkers connected B-cell epitopes with MHC class I epitopes and bridged MHC class II epitopes. Additionally, a rigid linker was placed between the adjuvant and B-cell epitopes to ensure adequate spatial separation (Figure 3).

The antigenicity of the final vaccine construct was evaluated, yielding a score of 0.8364, confirming its intrinsic antigenic potential. Allergenicity assessments classified the vaccine as non-allergenic, with no experimentally validated IgE-binding sites detected Table 8. Structural analysis of the vaccine construct predicted that 41.22% of the sequence consists of α-helices, contributing to structural stability, while 34.46% random coils provide flexibility for immune interactions. β-strands (19.59%) contribute to protein integrity, while 4.73% β-turns suggest regions that may facilitate immune receptor binding. The secondary structure predictions were consistent across different computational tools, supporting the structural feasibility of the vaccine construct.

**Table 8:** Multi-epitope vaccine candidate properties.

**Vaccine sequence**	**Length (AA)**	**Allergenicity^a^**	**Antigenicity score^b^**	**IgE epitope presence^c^**
AKFVAAWTLKAAAEAAAKFPDLLAGPGPGQIPHLAGTEQN FQGPGPGAQLNLIWQLAAYKYADKIYSIAAYFPGIYDALF AAYRSYRYKLLNWAAYNASLRLQRVRVADEGSGPGPGDRY VILGGHRDSGPGPGRSYRYKLLNWAYQQ	148	Non-allergen	0.8364	No experimentally validated IgE epitopes found

^a^AllerTOP v2.1 (https://www.ddg-pharmfac.net/allertop_test/).

^b^VaxiJen 2.0 (https://www.ddg-pharmfac.net/vaxijen/VaxiJen/VaxiJen.html).

^c^AlgPred (http://crdd.osdd.net/raghava/algpred/).

### 3.7 Modeling, 3D visualization, and validation of the vaccine structure

**Figure 4:**
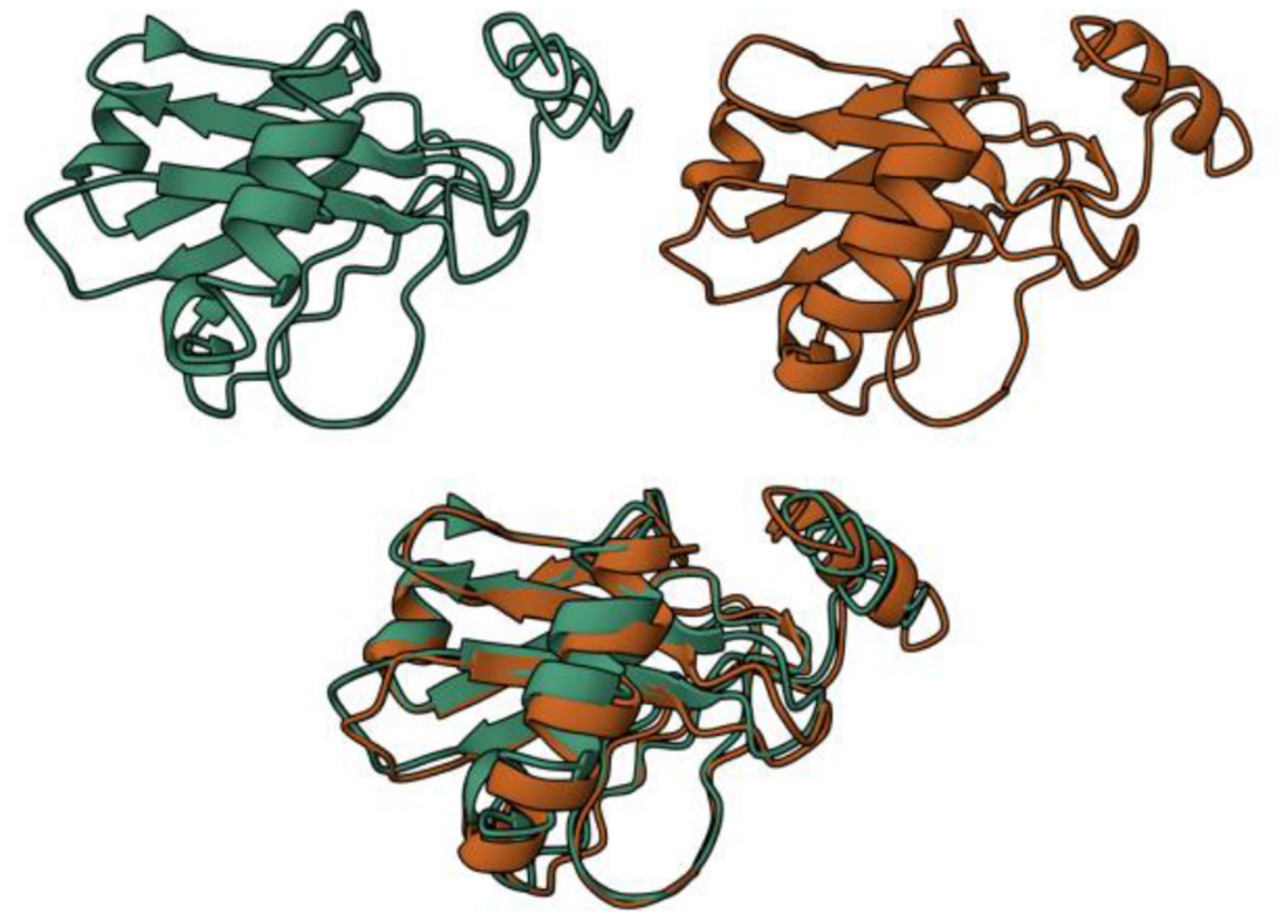
Visualization of the initial model (green) and the post-refinement model (brown).

After confirming the antigenicity, allergenicity, and secondary structure stability of the vaccine construct, 3D structural modeling and validation were performed to ensure its feasibility as a potential immunogen. This process involved homology-based structure prediction, model refinement, and structural assessment using computational tools. Five 3D structural models were generated, with C-scores ranging from −3.1 to −4.6. The C-score is a confidence measure derived from threading alignment significance, where higher values indicate better structural reliability. Model 1 was selected for further processing due to its highest C-score, suggesting superior template alignment. The root-mean-square deviation (RMSD) of the selected model was 12.0 ± 4.4 Å, and the Template Modeling (TM) score was 0.36 ± 0.12, indicating moderate structural similarity to known protein templates (Figure 4, green).

To enhance structural quality, the selected model was refined through computational optimization, improving backbone flexibility, steric interactions, and overall stability. Among the refined models, Model 3 was chosen based on its high residue accuracy and structural reliability (Figure 4, brown). The refined model demonstrated 91.1% of residues in Ramachandran favored regions and achieved a MolProbity score of 1.763, indicating a highly reliable structure with minimal steric clashes.

**Figure 5:**
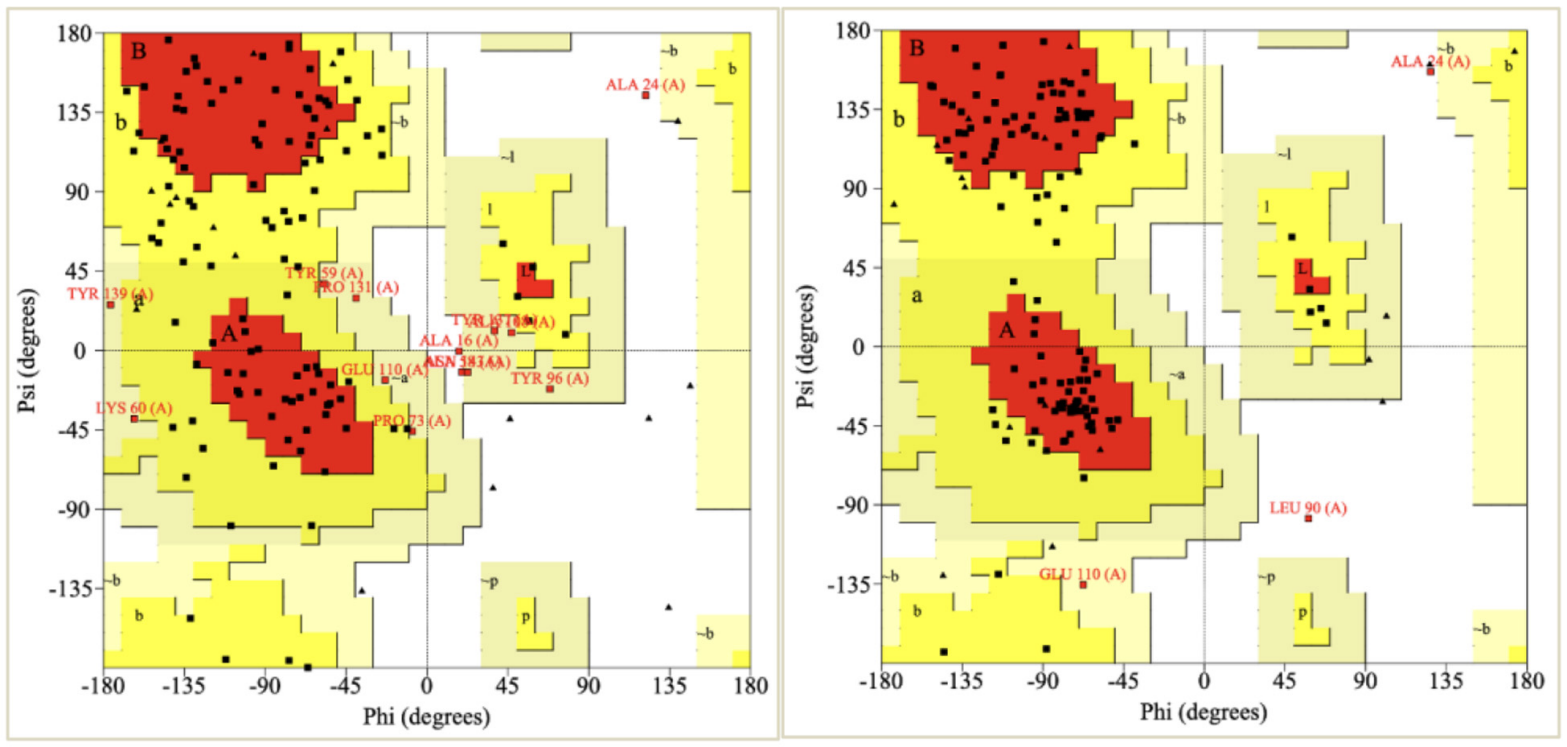
Ramachandran plot of the initial model **(left)** and the post-refinement model **(right)**.

Ramachandran plot analysis confirmed the improvement in structural accuracy after refinement (Figure 5). The refined model had 98.2% of residues in allowed regions, with 77.1% in the most favored regions, compared to 54.2% in the initial model. The percentage of residues in disallowed regions was reduced to 1.7% from 2.5%, indicating improved backbone geometry and steric compatibility.

**Figure 6:**
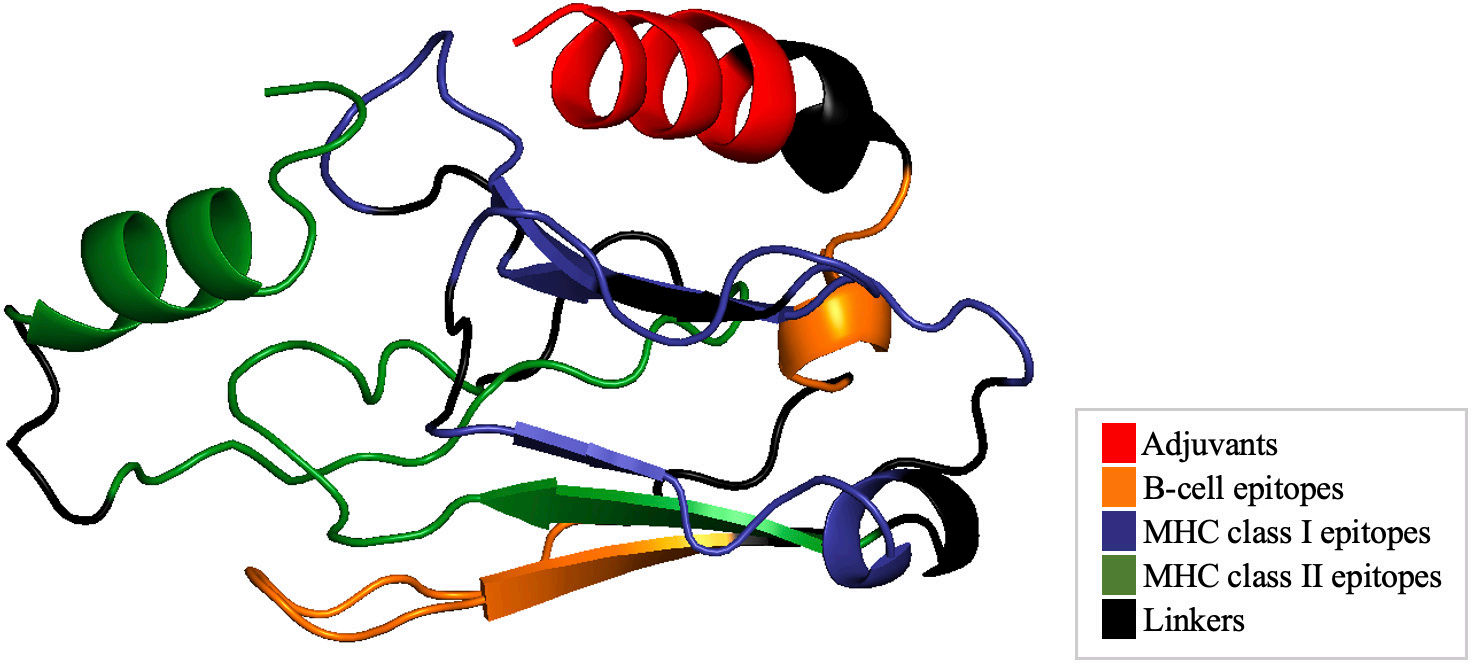
Three-dimensional structural representation of the final multi-epitope vaccine construct showing the spatial arrangement of functional domains, including adjuvants (red), linkers (black), B-cell epitopes (orange), MHC class I epitopes (green), and MHC class II epitopes (blue).

The spatial arrangement of functional domains was assessed through three-dimensional visualization of the final vaccine construct. The 3D model revealed the distribution of adjuvants (red), linkers (black), linear B-cell epitopes (orange), MHC class I epitopes (green), and MHC class II epitopes (blue), highlighting their integration into a compact and coherent framework. This visualization enabled detailed evaluation of epitope positioning and accessibility, suggesting favorable structural exposure for antigen recognition and immune receptor interaction (Figure 6).

### 3.8 Molecular docking and protein interaction analysis

**Figure 7:**
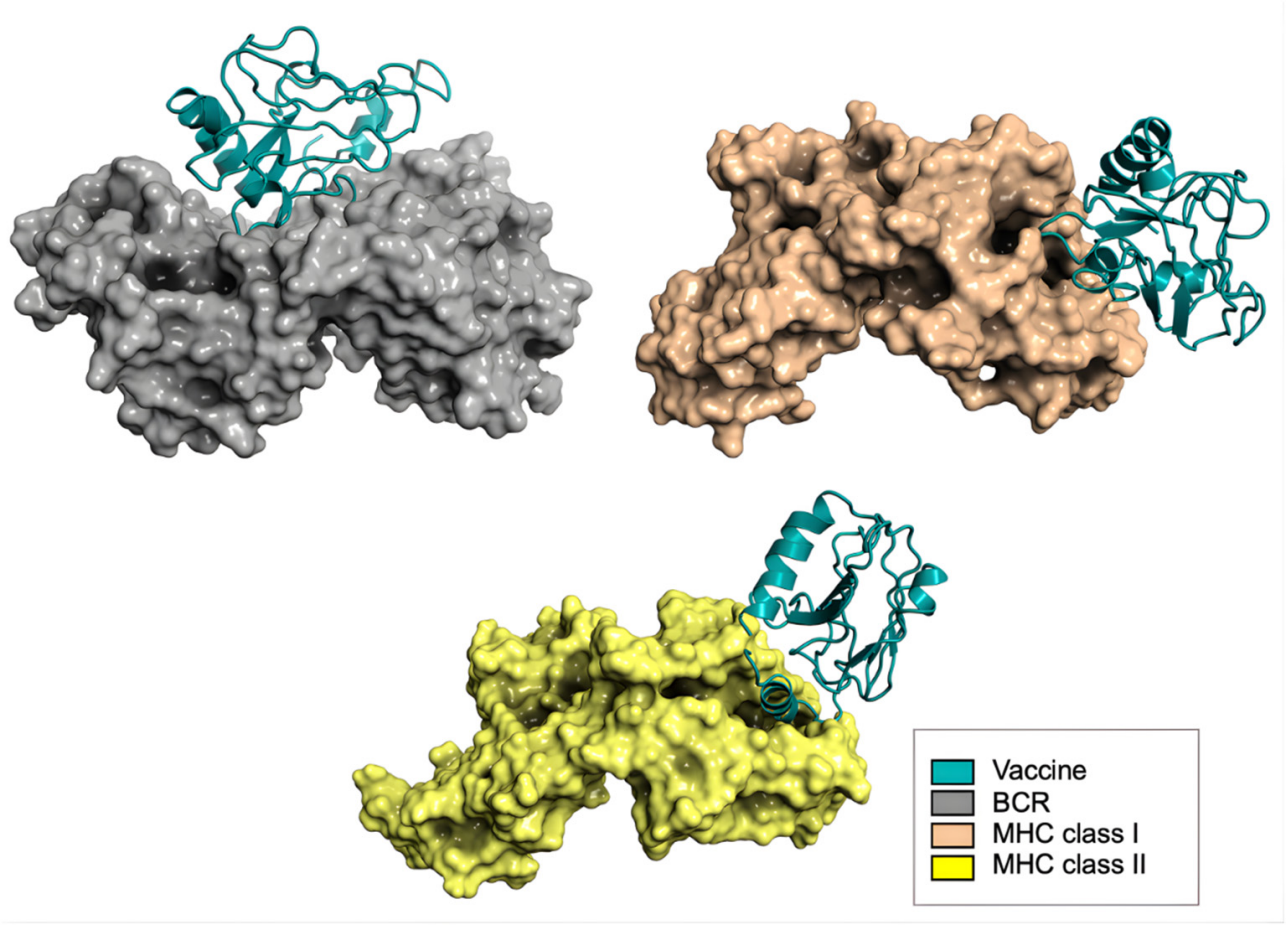
Molecular docking analysis of the multi-epitope vaccine construct (cyan) with key immune receptors, showing stable binding interactions with the B-cell receptor (gray), MHC class I (orange), and MHC class II (yellow).

Following structural validation of the vaccine construct, molecular docking was performed to evaluate its potential interactions with key adaptive immune receptors. Docking of the full construct with the B-cell receptor (BCR) demonstrated strong surface complementarity, indicating that the B-cell epitopes remained structurally accessible for antibody recognition. As illustrated in Figure 7, the vaccine (cyan) engaged the BCR (gray) in a stable orientation, supporting its capacity to elicit humoral responses.

For MHC class I (orange) and MHC class II (yellow) molecules, docking of the full construct was conducted only to provide a global visualization of receptor compatibility and to assess potential steric constraints. These poses were interpreted qualitatively, as physiological antigen presentation occurs through short peptide fragments that bind within the MHC groove rather than through full-length multi-epitope constructs. Functional assessment of antigen presentation was therefore based on epitope-level binding predictions performed earlier in the immunoinformatics workflow. Together, the BCR docking results and the epitope-specific MHC binding predictions support the capacity of the designed construct to engage both humoral and cellular immune pathways, reinforcing its candidacy as a precision immunotherapy approach for prostate cancer.

The docking scores were used to rank the stability of vaccine–receptor complexes. These scores represent an energy-weighted function incorporating electrostatic interactions, van der Waals forces, and desolvation effects, with lower values indicating more favorable conformations. The results revealed strong interactions between the vaccine construct and immune receptors (Table 9). The BCR complex showed the most favorable docking score (−962.3), followed by MHC class II (−926.4) and MHC class I (−799.7), suggesting a high potential for both B-cell and T-cell activation.

**Table 9:** Docking scores of the vaccine construct with BCR, MHC class I, and MHC class II receptors.

**Receptors**	**Docking score**
BCR (PDB ID: 5IFH)	−962.3
MHC I (PDB ID: 3BO8)	−799.7
MHC II (PDB ID: 4I5B)	−926.4

**Figure 8:**
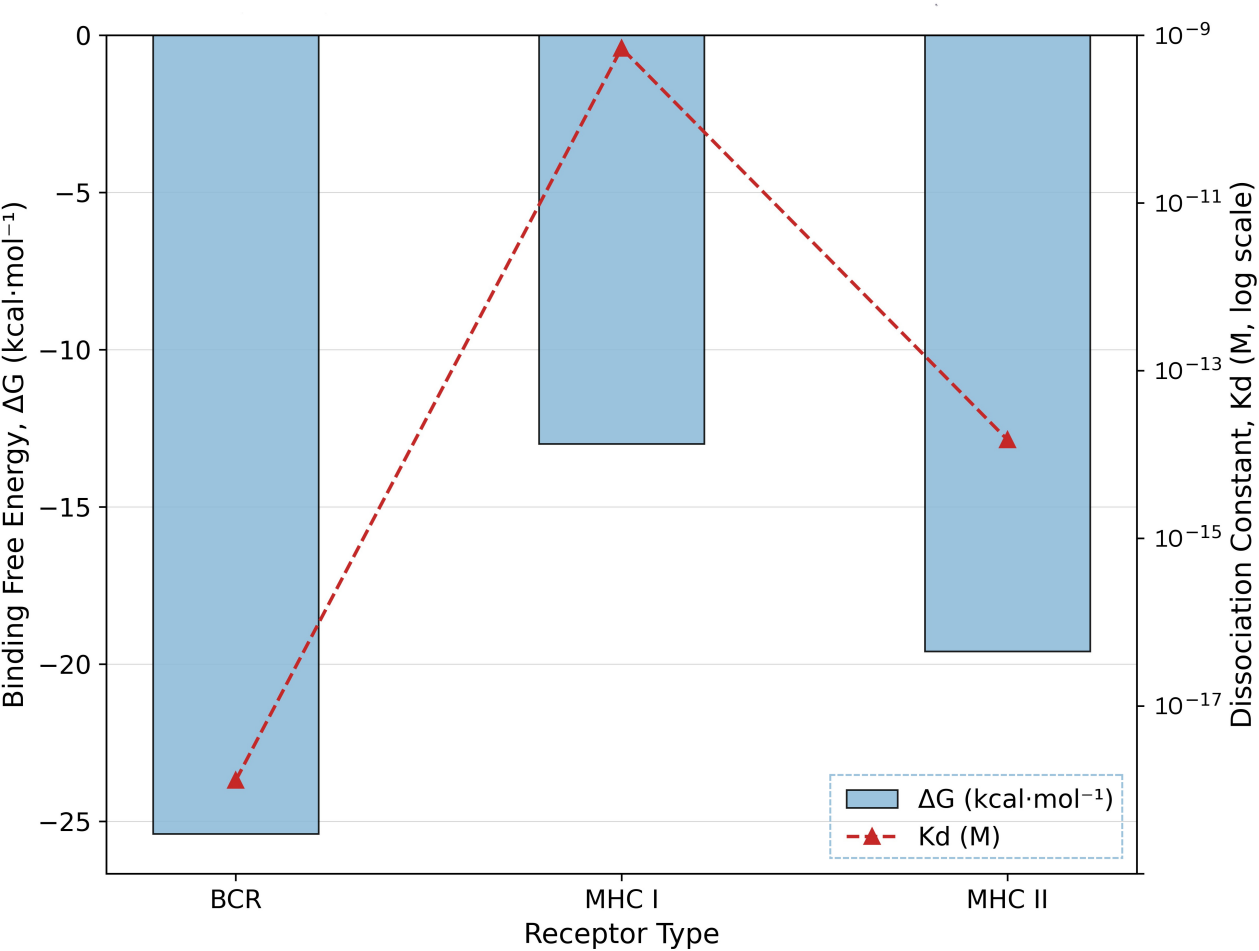
Binding-free energy (Δ*G*) and dissociation constant (*K*_d_) of peptide vaccine interactions with immune receptors.

Binding-free energy (Δ*G*) and dissociation constant (*K*_d_) were analyzed to validate interaction strength (Figure 8). The BCR–vaccine complex showed the strongest binding (Δ*G* = −25.4 kcal/mol; *K*_d_ = 1.3 × 10^−18^ M), followed by MHC class II (Δ*G* = −19.6 kcal/mol; *K*_d_ = 1.5 × 10^−14^ M). The MHC I–vaccine interaction was weaker (Δ*G* = −13.0 kcal/mol; *K*_d_ = 7 × 10^−10^ M), but remained within biologically relevant ranges. These differences indicate preferential binding of the vaccine to BCR and MHC II, supporting stronger recognition by these receptors compared to MHC I.

**Figure 9:**
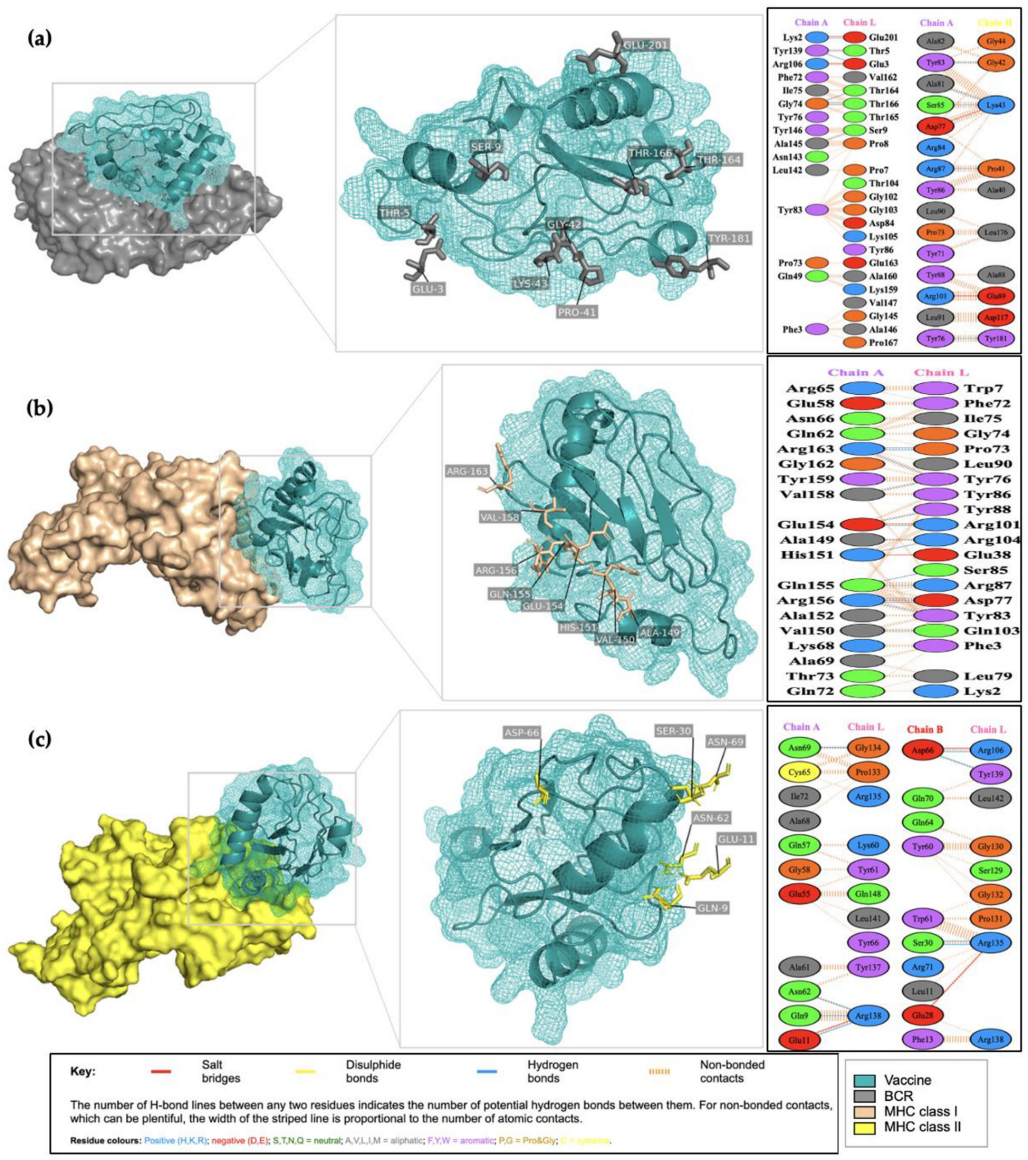
Visualization of molecular docking results and their interface interaction identification. **(a)** BCR-vaccine complex; **(b)** MHC I-vaccine complex; **(c)** MHC II-vaccine complex.

Analysis of vaccine-receptor complexes revealed stabilization primarily through hydrogen bonding, with additional contributions from salt bridges and hydrophobic interactions. Hydrogen bonds formed a stable interaction interface, facilitating ligand-receptor interactions and maintaining complex stability. The BCR-vaccine complex exhibited 12 hydrogen bonds, involving key residues in both the light and heavy chains (Figure 9a). For MHC class I, 14 hydrogen bonds within the peptide-binding groove (Figure 9b) contributed to stable and prolonged antigen presentation. The MHC class II complex exhibited 8 hydrogen bonds (Figure 9c), ensuring proper peptide orientation for effective antigen presentation to helper T cells. Alongside hydrogen bonding, additional electrostatic interactions and hydrophobic contacts contributed to peptide retention and antigen exposure. These interactions stabilized antigen binding, ensuring efficient immune cell recognition and activation.

### 3.9 Molecular dynamics simulation

**Figure 10:**
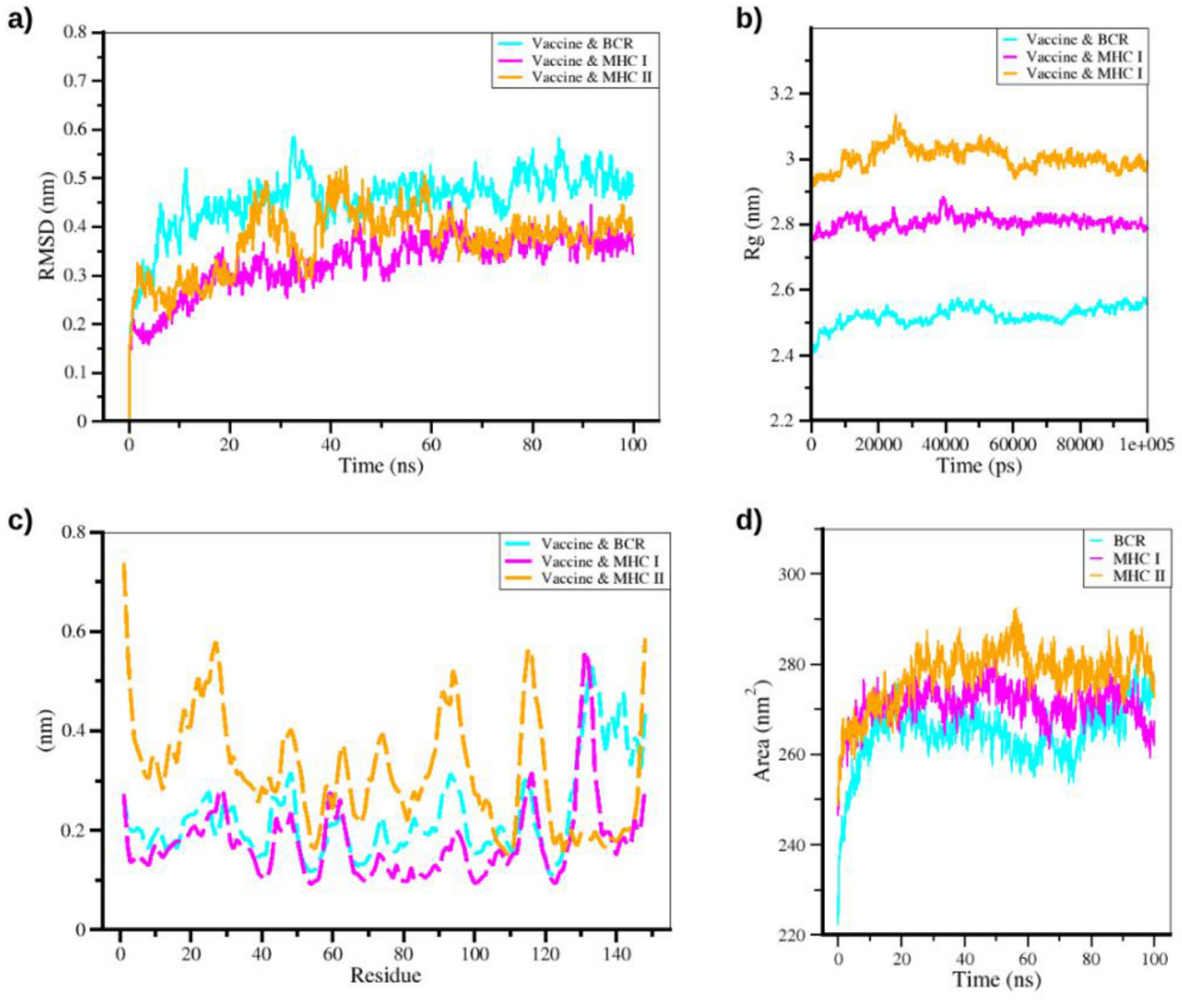
Graph of molecular dynamics analysis results for the binding complex with BCR, MHC I, and MHC II; **(a)** root mean square deviation; **(b)** radius of rotation; **(c)** root mean square fluctuation; **(d)** solvent accessible surface area.

Molecular dynamics simulations (MDS) were performed to evaluate the stability and dynamic behavior of the vaccine structure, while immune simulations were used to predict adaptive immune responses. These analyses provided insights into the construct's performance under simulated physiological conditions, with the results summarized in Figure 10. The vaccine–BCR complex exhibited structural stability as demonstrated by root-mean-square deviation (RMSD) analysis. After an initial rapid rise, the RMSD stabilized within 0.3–0.5 nm following early conformational adjustments, indicating overall equilibrium of the system. The vaccine–MHC I complex also displayed highly stable behavior. Radius of gyration (Rg) values confirmed compact tertiary structures across complexes, with the vaccine–BCR complex fluctuating around 2.48 nm, the vaccine–MHC I complex around 2.79 nm, and the vaccine–MHC II complex around 2.91 nm, the latter showing relatively higher flexibility.

Residue-wise root-mean square fluctuation (RMSF) analysis revealed minimal fluctuation in the protein core, while regions around residues 100–110 and 140–150 displayed higher mobility, consistent with dynamic functional domains. Solvent-accessible-surface area (SASA) values for all complexes fluctuated between 220 and 290 nm^2^, reflecting dynamic surface exposure important for molecular interactions and antigen recognition. These MDS results emphasize the structural integrity and necessary conformational adaptability of the vaccine structure under simulated physiological conditions.

### 3.10 Immune simulation analysis

Immune simulation results are provided in the Supplementary material.

## 4 Discussion

Prostate cancer remains a major global health concern, necessitating the development of novel immunotherapeutic strategies that effectively target tumor-associated antigens. In this study, an immunoinformatics-driven approach was employed to design a multi-epitope peptide-based vaccine targeting B7H3, PSMA, and STEAP1, three tumor-associated antigens known for their roles in prostate cancer progression (82, 83), immune evasion (27, 84, 85), and modulation of the tumor microenvironment (86–89). By incorporating epitopes from multiple antigens, this vaccine aims to overcome tumor heterogeneity and reduce the likelihood of immune escape (16). This design reflects a precision medicine framework by leveraging well-characterized molecular biomarkers to generate targeted immune responses and maximizing potential therapeutic benefit across diverse patient populations.

Antigen selection in this study was based on published evidence demonstrating high expression of PSMA, STEAP1, and B7-H3 in prostate cancer (19–23), no new transcriptomic analysis of patient cohorts (e.g., TCGA) was performed, as such datasets primarily capture gene-level expression and do not directly validate epitope-level immunogenicity. Accordingly, the biological relevance of the selected epitopes will require confirmation through targeted *in vitro* and *in vivo* assays, including antigen presentation and T-cell activation studies, as a necessary next step toward translational evaluation (90–92). In addition, dedicated co-expression profiling across large patient cohorts would further strengthen clinical relevance by refining estimates of the patient populations most likely to benefit from a multi-antigen vaccine strategy.

Although PSMA, STEAP1, and B7H3 were prioritized due to their strong tumor specificity, consistent overexpression, well-defined immunogenic domains, and established translational relevance, we acknowledge that other prostate cancer–associated antigens (including TROP2, DLL3, PSCA, and PAP, PSA, NY-ESO-1, and SSX-2) may also offer therapeutic value. Expanding the antigen panel and systematically comparing candidates within the same immunoinformatics pipeline represents an important direction for future work, particularly to assess whether additional or higher-scoring antigen combinations could further enhance vaccine breadth, immunogenic performance, and resistance to immune escape. However, antigen expansion must be carefully balanced against constraints on vaccine construct length, as excessively large constructs can impair antigen processing efficiency, increase epitope competition, and reduce manufacturability (93, 94). For this reason, the current design intentionally focuses on a compact set of high-value antigens while acknowledging that broader antigen comparison remains an important direction for future optimization.

Multi-epitope strategies have been explored in cancer vaccine research, demonstrating enhanced immune activation and reduced tumor resistance compared to single-antigen approaches (16, 95). The immunogenic epitopes in this study were carefully selected based on antigenicity, toxicity, and global population coverage. B-cell epitopes were chosen to elicit humoral immunity, promoting antibody-mediated tumor cell elimination, while T-cell epitopes were selected for their strong binding affinity to MHC class I and II molecules to ensure robust activation of CD8^+^ cytotoxic T cells and CD4^+^ helper T cells. The inclusion of PADRE as an adjuvant further enhanced immunogenicity by facilitating antigen presentation (96–99). Population coverage analysis revealed that the selected epitopes provided 97.51% global coverage, ensuring broad immunological protection across diverse HLA alleles. Such broad coverage improves vaccine applicability across populations and enhances accessibility compared with individualized immunotherapies such as Sipuleucel-T (100).

Molecular docking and interaction analyses demonstrated that the vaccine construct formed highly stable complexes with key immune receptors, particularly the BCR and MHC class II molecules, indicating strong potential to stimulate both humoral and cellular immune responses. Preferential binding to BCR suggests robust activation of B-cell–mediated immunity, while stable interactions with MHC molecules support efficient antigen presentation to T cells. The predominance of hydrogen bonding, complemented by salt bridges and hydrophobic contacts, highlights the structural robustness of these complexes. Collectively, these findings align with previous reports demonstrating that stable epitope-receptor interactions are critical for durable immunogenicity and effective vaccine performance, reinforcing the construct's promise as a precision immunotherapy candidate for prostate cancer (101). In line with the epitope-centric design framework of the present study, docking analyses were intentionally focused on the B-cell receptor to evaluate antigen recognition and adaptive immune engagement. Docking with Toll-like receptors was not pursued, as innate immune activation mechanisms were beyond the primary scope of this work. Nevertheless, complementary evaluation of innate immune receptor interactions, including Toll-like receptor engagement, may further inform vaccine-host interactions and represents an important direction for future investigation.

Hydrogen bonding has long been recognized as a key determinant of ligand–receptor stability, providing directional interactions with minimal energy requirements and playing an essential role in catalytic and genetic regulation (102, 103). Previous studies have emphasized that, among various chemical interactions, hydrogen bonds are unique in creating directional interactions with minimal energy requirements, thereby playing an essential role in regulating genetic and catalytic functions (74). Consistent with these observations, hydrogen bonding emerged as the predominant stabilizing force in the vaccine–receptor complexes.

In the BCR complex, the extensive hydrogen bond network is likely to support B-cell activation and subsequent antibody generation (104). Within the MHC class I complex, hydrogen bonds located in the peptide-binding groove may contribute to stable antigen retention and prolonged presentation to cytotoxic T cells (105). Similarly, in the MHC class II complex, hydrogen bonds appeared to stabilize the peptide for efficient antigen presentation to CD4^+^ helper T cells (106–108). Beyond hydrogen bonding, electrostatic interactions and hydrophobic contacts provided additional stability to the vaccine–receptor interface (109). Together, these non-covalent forces promote a well-defined conformation that favors immune recognition and supports the construct's potential as a precision immunotherapy candidate.

Molecular dynamics simulation further confirmed the structural stability of the vaccine construct under physiological conditions. RMSD analysis demonstrated gradual stabilization with only minor conformational adjustments, indicating that the construct maintained overall equilibrium (110). The radius of gyration and solvent-accessible surface area analyses showed that the vaccine preserved a compact tertiary structure with limited fluctuations (111, 112), supporting its structural robustness. The observed reduction in hydrogen bonds is consistent with natural molecular rearrangements during simulation and does not detract from overall structural stability.

Immune simulation results indicated that the vaccine candidate successfully induced strong and durable adaptive immune responses. The rise in Ig titers following antigen clearance suggested effective immune priming, and the persistence of memory B cells beyond 100 days signaled long-lasting protection. T-cell responses were marked by expansion of CD8^+^ cytotoxic and CD4^+^ helper T cells, supporting durable anti-tumor immunity. Cytokine patterns further reinforced these findings. Increases in IL-2, IFN-γ, and IL-12 corresponded to enhanced T-cell proliferation, cytotoxicity, and antigen presentation (113–118), whereas regulatory cytokines such as TGF-β and IL-10 suggested physiological immune balancing rather than vaccine-induced tolerance (119).

Compared to existing prostate cancer vaccines, this multi-epitope construct offers several advantages. Multi-epitope vaccines have been shown to stimulate broader and more sustained immune responses than single-antigen approaches (120). By targeting multiple tumor-associated antigens, the likelihood of immune evasion is reduced, which is particularly important for heterogeneous cancers such as prostate cancer. Unlike Sipuleucel-T, which requires individualized autologous cell processing, this peptide-based vaccine can be mass-produced through a cost-effective and scalable platform (94). Peptide vaccines also avoid vector-associated risks and offer simpler storage and distribution than viral or mRNA-based formats (95, 121). The multi-epitope design therefore enables simultaneous targeting of multiple tumor antigens, a strategy that may improve therapeutic efficacy while limiting the potential for immune escape (16).

Despite the promising *in silico* results, several limitations must be acknowledged before clinical translation. Each computational approach carries inherent constraints, requiring cautious interpretation. While molecular docking and immune simulations predict favorable interactions, experimental validation is necessary to confirm these findings in biological systems. Docking analyses, for example, do not fully account for dynamic conformational changes *in vivo* and may overestimate binding affinity (122, 123). MDS provide valuable insights into structural stability but are restricted by simulation time frames and may not capture the full complexity of physiological conditions (124, 125). Thus, stability inferred from MD trajectories may not fully reflect biological behavior and requires confirmation through *in vitro* stability assays, proteolytic susceptibility testing, and biophysical characterization.

Similarly, immune simulations offer useful predictions but cannot reproduce the complexity of the tumor microenvironment. Immune suppression, stromal interactions, hypoxia, and cytokine gradients critically shape anti-tumor responses but are not fully captured by current *in silico* models. Simulation-derived cytokine or T-cell activation patterns should therefore be interpreted as hypothesis-generating rather than definitive predictors of efficacy. Future validation in *ex vivo* human immune assays and *in vivo* tumor-bearing models will be essential. Furthermore, multi-epitope vaccines intrinsically face challenges such as potential epitope competition, suboptimal processing of individual epitopes, and unpredictable immunodominance hierarchies. The computational pipeline mitigated these challenges by applying antigenicity screening, conservancy analysis, structural modeling, and population coverage filtering, yet *in silico* selection cannot fully eliminate these risks.

To bridge the gap between computational predictions and experimental outcomes, comprehensive *in vitro* and *in vivo* validation is required. *In vitro* studies should include T-cell activation assays to evaluate cellular responses, along with *ex vivo* experiments using patient-derived immune cells to confirm antigen presentation and immune activation. *In vivo* testing in immunocompetent murine models would further assess vaccine efficacy and characterize immune infiltration into the tumor microenvironment (126, 127). Given that Southeast Asia and South Africa exhibited lower HLA representation, region-specific epitope optimization may be needed to enhance vaccine inclusivity. Although all epitopes were derived from prostate cancer–associated antigens, a proteome-wide similarity assessment to exclude unintended matches with human proteins remains an essential future step to ensure safety and specificity. Additionally, immunoinformatics-based predictions cannot fully replicate the complexity of *in vivo* immune responses. Accordingly, future work will employ multi-algorithm cross-validation and targeted experimental assays, including *in vitro* T-cell activation and *in vivo* immunogenicity testing, to refine epitope performance and improve the reliability of computational predictions.

This study underscores the advantages of an immunoinformatics-driven approach in accelerating vaccine design by reducing the time and cost associated with conventional methods. Computational strategies enable precise selection of immunogenic epitopes with broad HLA coverage, while the integration of multiple tumor-associated antigens enhances immune breadth and reduces the likelihood of immune escape. The resulting multi-epitope construct aligns with precision medicine principles by leveraging prostate cancer–associated biomarkers (B7-H3, PSMA, and STEAP1) to elicit targeted immune responses. Although further experimental validation is required, the findings provide a strong foundation for preclinical development and support the potential of a scalable, cost-effective, and precision-oriented peptide vaccine for prostate cancer.

## 5 Conclusions

This study successfully designed a multi-epitope peptide vaccine candidate against prostate cancer through an immunoinformatics-driven approach. The construct demonstrated strong antigenicity, structural stability, and the potential to elicit robust adaptive immune responses, providing a solid foundation for further validation. Future work should include peptide synthesis, *in vitro* T-cell activation assays, and dendritic cell presentation studies, followed by preclinical evaluation in immunocompetent models to confirm tumor-specific responses and *in vivo* efficacy. For clinical translation, key challenges such as regulatory approval, preclinical safety testing, and early-phase trial design must be addressed to ensure safety, immunogenicity, and efficacy in patients. Integration with immune checkpoint inhibitors may further enhance therapeutic benefit by counteracting tumor-induced immune suppression. Importantly, this vaccine reflects the principles of precision medicine by leveraging prostate cancer–associated biomarkers (B7-H3, PSMA, and STEAP1) to generate tailored immune responses capable of addressing tumor heterogeneity and reducing the risk of immune escape. With further validation, this multi-epitope construct represents a promising and scalable immunotherapeutic strategy that could improve patient outcomes and expand treatment options in prostate cancer.

## Data Availability

The protein sequences analyzed in this study were retrieved from publicly available databases, including the NCBI GenBank database (accession numbers NP_036581.1 and AAA60209.1) and the UniProt database (accession number Q5ZPR3). All data generated or analyzed during this study are included in this published article and its Supplementary material.
