# Correction: Immunoinformatics-driven multi-epitope vaccine design targeting PSMA, STEAP1, and B7H3 for prostate cancer

**DOI:** 10.3389/fmed.2026.1824612

**Published:** 2026-05-08

**Authors:** Stefanus Vicky Bernhard Elisa Runtunuwu, Trina Ekawati Tallei, Hyo Jeong Kim, Moon Nyeo Park, Ismail Celik, Burak Kirilmaz, Grace Lendawati Amelia Turalaki, Fatimawali Fatimawali, Lydia Estelina Naomi Tendean, Martha Marie Kaseke, Dionisius Rafael Makawaehe, Elne Vieke Rambi, Bonglee Kim

**Affiliations:** 1 Medical Education Study Program, Faculty of Medicine, Sam Ratulangi University, Manado, Indonesia; 2 Department of Biology, Faculty of Medicine, Sam Ratulangi University, Manado, Indonesia; 3 Department of Biology, Faculty of Mathematics and Natural Sciences, Sam Ratulangi University, Manado, Indonesia; 4 Department of Pathology, College of Korean Medicine, Kyung Hee University, Seoul, Republic of Korea; 5 Korean Medicine-Based Drug Repositioning Cancer Research Center, College of Korean Medicine, Kyung Hee University, Seoul, Republic of Korea; 6 Department of Pharmaceutical Chemistry, Faculty of Pharmacy, Erciyes University, Kayseri, Türkiye; 7 Pharmacy Study Program, Faculty of Mathematics and Natural Sciences, Sam Ratulangi University, Manado, Indonesia; 8 Department of Anatomy and Histology, Faculty of Medicine, Sam Ratulangi University, Manado, Indonesia; 9 Department of Medical Laboratory Technology, Politeknik Kesehatan Kemenkes Manado, Manado, Indonesia

**Keywords:** computational vaccine design, immunoinformatics, immunotherapy, multi-epitope, precision medicine, prostate cancer, tumor-associated antigens

In the published article, there was an inconsistency between the antigenicity score reported in the text and the value shown in Table 8.

A correction has been made to the section **3 Results**, *3.6 Multi-epitope vaccine construction and analysis of antigenicity, allergenicity, and secondary structure of the vaccine candidate*, Paragraph 2:

“The antigenicity of the final vaccine construct was evaluated, yielding a score of 0.9093, confirming its intrinsic antigenic potential.”

This sentence has been revised to read:

“The antigenicity of the final vaccine construct was evaluated, yielding a score of 0.8364, confirming its intrinsic antigenic potential.”

In the published article, there was a typographical duplication in the sentence describing the molecular dynamics methods.

A correction has been made to the section **2 Materials and methods**, *2.9 Molecular dynamics simulation*, Paragraph 1:

“Molecular dynamics simulation (MDS) was performed to analyze the interactions of epitopes with MHC class I, MHC class II, and B cell receptors (BCR) under physiological conditions. The simulation was performed using was prepared using the CHARMM-GUI online server due to its compatibility with efficient protein-water simulations and standard force fields, and the simulations were run using AMBER ff14SB force fields in GROMACS version 2023 (GROMACS Development Team, Stockholm, Sweden) (67).”

This sentence has been revised to read:

“The system was prepared using the CHARMM-GUI online server due to its compatibility with efficient protein-water simulations and standard force fields, and simulations were performed using the AMBER ff14SB force fields in GROMACS version 2023 (GROMACS Development Team, Stockholm, Sweden) (67).”

In the published article, a sentence in the Discussion was duplicated consecutively.

A correction has been made to the section **4 Discussion**, Paragraph 9:

“The rise in Ig titers following antigen clearance suggested effective priming of the immune system. The rise in Ig titers following antigen clearance suggested effective immune priming, and the persistence of memory B cells beyond 100 days signaled long-lasting protection.”

This has been revised to read:

“The rise in Ig titers following antigen clearance suggested effective immune priming, and the persistence of memory B cells beyond 100 days signaled long-lasting protection.”

In the published article, there was a typographical error in the Discussion section.

A correction has been made to the section **4 Discussion**, Paragraph 9:

“Cytokine patterns further reinforced these findings. Increases in IL-2, IFN-γ, and IL-12 corresponded to enhanced T-cell proliferation, cytotoxicity, and antigen presentation (113–118), whereas regulatory cytokines such as TGF-β and IL-10 suggested physiological immune balancing rather than vaccine-induced tolerancee (119).”

This has been revised to read:

“Cytokine patterns further reinforced these findings. Increases in IL-2, IFN-γ, and IL-12 corresponded to enhanced T-cell proliferation, cytotoxicity, and antigen presentation (113–118), whereas regulatory cytokines such as TGF-β and IL-10 suggested physiological immune balancing rather than vaccine-induced tolerance (119).

The original version of this article has been updated.

