# Supplementary material for: Immunoinformatics-driven multi-epitope vaccine design targeting PSMA, STEAP1, and B7H3 for prostate cancer
Source: Front Med (Lausanne). 2026 Mar 5;13:1716345. doi: 10.3389/fmed.2026.1716345 (PMC12999402; doi:10.3389/fmed.2026.1716345)
Supplement: Supplementary file 1 [file Data_Sheet_1.pdf]

Supplementary Materials: Immune Simulation Analysis

Immune simulation analysis provided insights into the adaptive immune responses elicited by the vaccine candidate. Antigen clearance was accompanied by a marked rise in antibody titers, including IgM, IgG1, and IgG2, demonstrating the ability of the construct to induce a strong humoral response, a critical factor for long-term immunity (**Figure 11a**). The total B-cell population increased sharply after immunization, peaking around day 40 before gradually declining, while maintaining a stable pool of memory B cells. This profile indicates effective induction of a primary humoral response and the establishment of long-term immunological memory, ensuring durable protection. The persistence of memory B cells beyond 100 days suggests readiness for rapid recall upon antigen re-exposure.

Helper T cells (Th) also expanded rapidly following each antigen injection, reflecting their role in coordinating B-cell activation and cytotoxic T-cell responses. Correspondingly, the population of active cytotoxic T cells (CD8+) increased significantly, confirming the construct's potential to stimulate effective elimination of target cancer cells (**Figure 11c–e**). Importantly, the frequency of anergic cells across all subsets remained low, indicating that the vaccine efficiently activated immune pathways without promoting tolerance or immunosuppression (**Figure 11d, 11f**).

The cytokine profile (**Figure 12**) further validated the vaccine's immunogenic potential. The significant surge in IL-2 secretion confirmed effective T-cell activation and proliferation. Elevated IFN-ɣ levels were also observed, highlighting its role in strengthening both innate and adaptive immune responses. IL-12 was markedly upregulated, supporting the activation of cytotoxic T cells and natural killer (NK) cells. In addition, the detection of regulatory cytokines such as TGF-β and IL-10 indicated the presence of mechanisms that balance immune activation and limit excessive responses. Collectively, these findings demonstrate that the multi-epitope peptide vaccine elicited a robust adaptive immune response against prostate cancer. The combined activation of B cells, helper T cells, and $CD8^+$ T cells, together with a favorable cytokine milieu, suggests the potential of this construct to induce effective tumor-targeted immunity.

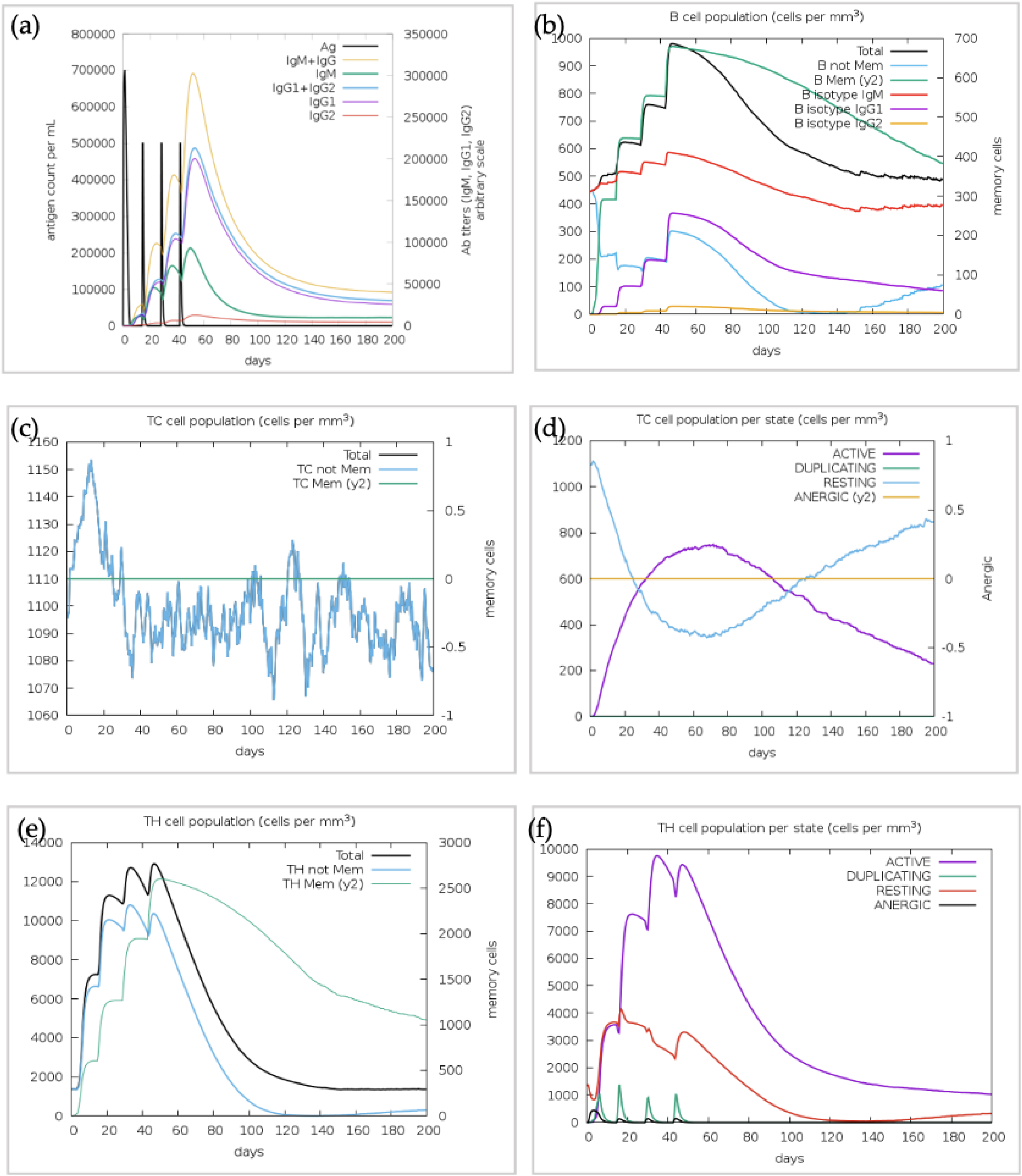

Figure 11. Immune simulation analysis of the multi-epitope vaccine construct showing (a) antigen clearance and antibody responses (IgM, IgG1, IgG2), (b) B-cell population dynamics and memory formation, (c) helper T-cell (Th) activation, (d) cytotoxic T-cell (CD8+) responses, (e) reduction of antigen load, and (f) low frequency of anergic cells, confirming effective immune activation without tolerance induction.

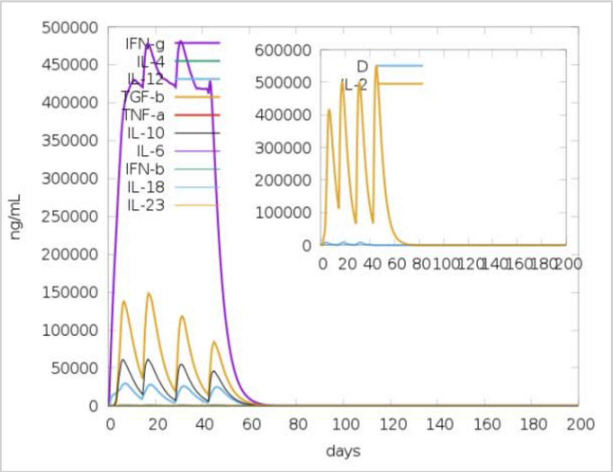

Figure 12. Cytokine profile from immune simulation of the multi-epitope vaccine construct, showing elevated IL-2, IFN-ɣ, and IL-12 levels associated with T-cell activation and cytotoxic responses, alongside regulatory cytokines (TGF-β and IL-10) that balance immune activation.
